# Single-cell RNA sequencing uncovers the nuclear decoy lincRNA PIRAT as a regulator of systemic monocyte immunity during COVID-19

**DOI:** 10.1073/pnas.2120680119

**Published:** 2022-08-23

**Authors:** Marina Aznaourova, Nils Schmerer, Harshavardhan Janga, Zhenhua Zhang, Kim Pauck, Judith Bushe, Sarah M. Volkers, Daniel Wendisch, Philipp Georg, Evgenia Ntini, Michelle Aillaud, Margrit Gündisch, Elisabeth Mack, Chrysanthi Skevaki, Christian Keller, Christian Bauer, Wilhelm Bertrams, Annalisa Marsico, Andrea Nist, Thorsten Stiewe, Achim D. Gruber, Clemens Ruppert, Yang Li, Holger Garn, Leif E. Sander, Bernd Schmeck, Leon N. Schulte

**Affiliations:** ^a^Institute for Lung Research, Philipps University Marburg, 35043 Marburg, Germany;; ^b^Department of Computational Biology for Individualised Medicine, Centre for Individualised Infection Medicine & TWINCORE, joint ventures between the Helmholtz-Centre for Infection Research and the Hannover Medical School, 30625 Hannover, Germany;; ^c^Department of Genetics, University of Groningen and University Medical Center Groningen, 9713 AV, Groningen, The Netherlands;; ^d^Translational Inflammation Research Division & Core Facility for Single Cell Multiomics, Philipps University Marburg, 35043 Marburg, Germany;; ^e^Institute of Veterinary Pathology, Freie Universitaet Berlin, 14195 Berlin, Germany;; ^f^Department of Infectious Diseases and Respiratory Medicine, Charité-Universitätsmedizin Berlin, Corporate Member of Freie Universität Berlin, Humboldt-Universität zu Berlin, and Berlin Institute of Health, 10117 Berlin, Germany;; ^g^Max Planck Institute for Molecular Genetics, 14195 Berlin, Germany;; ^h^Institute of Molecular Biology and Biotechnology, FORTH, Heraklion, GR-70013, Greece;; ^i^Institute of Laboratory Medicine, Philipps University Marburg, 35043 Marburg, Germany;; ^j^Department of Hematology, Oncology and Immunology, Philipps University Marburg, University Hospital Giessen and Marburg, 35043 Marburg, Germany;; ^k^Universities of Giessen and Marburg Lung Center (UGMLC), Giessen, 35392 Germany;; ^l^German Center for Lung Research (DZL), Giessen, 35392 Germany;; ^m^Institute of Virology, University Hospital Giessen and Marburg, 35043 Marburg, Germany;; ^n^Department of Gastroenterology, Endocrinology, Metabolism and Infectiology, University Hospital of Giessen and Marburg, Philipps University Marburg, 35043 Marburg, Germany;; ^o^Institute for Computational Biology, Helmholtz Centre, 85764 Munich, Germany;; ^p^Genomics Core Facility, Philipps University Marburg, 35043 Marburg, Germany;; ^q^Institute of Molecular Oncology, Philipps University Marburg, 35043 Marburg, Germany;; ^r^UGMLC Giessen Biobank and european IPF registry (eurIPFreg), Giessen, 35392 Germany;; ^s^Department of Internal Medicine, Radboud Institute for Molecular Life Sciences, Radboud University Medical Center, 6525 GA Nijmegen, The Netherlands;; ^t^Department of Respiratory and Critical Care Medicine, University Medical Center Marburg, 35043 Marburg, Germany;; ^u^Center for Synthetic Microbiology, Philipps University Marburg, 35043 Marburg, Germany;; ^v^German Center of Infection Research, 35043 Marburg, Germany

**Keywords:** COVID-19, single-cell RNA-seq, long noncoding RNA, PU.1, immunity

## Abstract

The systemic immune response to viral infection is shaped by master transcription factors, such as NF-κB, STAT1, or PU.1. Although long noncoding RNAs (lncRNAs) have been suggested as important regulators of transcription factor activity, their contributions to the systemic immunopathologies observed during SARS-CoV-2 infection have remained unknown. Here, we employed a targeted single-cell RNA sequencing approach to reveal lncRNAs differentially expressed in blood leukocytes during severe COVID-19. Our results uncover the lncRNA PIRAT (PU.1-induced regulator of alarmin transcription) as a major PU.1 feedback-regulator in monocytes, governing the production of the alarmins S100A8/A9, key drivers of COVID-19 pathogenesis. Knockout and transgene expression, combined with chromatin-occupancy profiling, characterized PIRAT as a nuclear decoy RNA, keeping PU.1 from binding to alarmin promoters and promoting its binding to pseudogenes in naïve monocytes. NF-κB–dependent PIRAT down-regulation during COVID-19 consequently releases a transcriptional brake, fueling alarmin production. Alarmin expression is additionally enhanced by the up-regulation of the lncRNA LUCAT1, which promotes NF-κB–dependent gene expression at the expense of targets of the JAK-STAT pathway. Our results suggest a major role of nuclear noncoding RNA networks in systemic antiviral responses to SARS-CoV-2 in humans.

## Significance

SARS-CoV-2–infected patients often display characteristic changes in the production of immune mediators that trigger life-threatening courses of COVID-19. The underlying molecular mechanisms are not yet fully understood. Here, we used single-cell RNA sequencing to investigate the involvement of the emerging class of long regulatory RNA in COVID-19. Our data reveal that a previously unknown regulatory RNA in the nucleus of immune cells is altered after SARS-CoV-2 infection. The degradation of this RNA removes a natural brake on the production of critical immune mediators that can promote the development of severe COVID-19. We believe that therapeutic intervention in this nuclear RNA circuit could counteract the overproduction of disease-causing immune mediators and protect against severe COVID-19.

Severe courses of infection often culminate in deregulated host responses, ranging from overproduction of inflammation mediators to immune-paralysis (1, 2). During infections with the pandemic coronavirus SARS-CoV-2, elevated serum levels of NF-κB–dependent proinflammatory interleukins (IL) repeatedly coincide with deranged type I interferon (IFN) immunity and signs of immune-exhaustion (3), rendering host-directed therapies a complex effort (4). Single-cell RNA sequencing (scRNA-seq) of peripheral blood mononuclear cells (PBMCs) from patients with coronavirus disease 2019 (COVID-19) additionally uncovered a dysregulated myeloid compartment, comprising monocytes and granulocytes. Whereas in patients with mild COVID-19 an increase in activated classic (CD14^+^) monocytes is observed, severe COVID-19 is marked by the accumulation of dysfunctional classic monocytes with reduced HLA-DR expression and immature neutrophils (5, 6). Additionally, a reduction in nonclassic (CD16^high^) monocytes has been observed (5, 6). Overt production of the alarmins S100A8 and S100A9 by monocytes and neutrophils appears to be involved in these alterations (5–7). The nuclear circuits driving these complex immune rearrangements remain poorly understood.

Myeloid immune cell activation during infection largely relies on the sensing of pathogen associated molecular patterns (PAMPs) and soluble immune mediators through dedicated receptors. Examples are Toll-like receptor 3 (TLR3), a sensor of viral double-stranded RNA, and TLR4, which senses bacterial lipopolysaccharide (LPS) (8). TLR4 and other PAMP and cytokine receptors activate the proinflammatory master transcription factor NF-κB through the MyD88-dependent signaling cascade. TLR3 activates the TRIF-dependent signaling cascade, which may also be activated by TLR4. In myeloid cells, TRIF-signaling results in IRF3 transcription factor activation and production of type I IFNs. The latter stimulate JAK-STAT–dependent antiviral responses (8). To counteract misguided leukocyte responses, mammalian immune systems have evolved sophisticated mechanisms, keeping immune gene expression within tight limits. Examples are immune-modulatory splice-regulators, such as the SF3B snRNP (9, 10), or regulators of signaling complex assembly, such as Optineurin (11). Besides proteins, long-noncoding RNAs (lncRNAs) are increasingly recognized as regulators of mammalian immune responses. Defined as noncoding transcripts ≥200 nts, lncRNAs constitute a heterogeneous category of RNA, participating in protein complex assembly, disintegration, and turnover (12–14). So far, only a minor fraction of the ∼20,000 human lncRNAs has been characterized and their roles in the human immune system are only beginning to be explored (14). Among the few characterized lncRNAs in this context is MaIL1, which associates with the ubiquitin-reader OPTN to promote TBK1–dependent IRF3 phosphorylation, and thus type I IFN immunity (14). GAPLINC, PACER, and CARLR regulate proinflammatory gene expression by adjusting NF-κB p50/p65 expression and activity (15–17). Despite the emerging roles of noncoding RNAs in immunity, however (18), the exploration of lncRNA mechanisms contributing to severe COVID-19 has lagged behind.

Here, we used scRNA-seq to study lncRNAs involved in the systemic immunopathologies during COVID-19. Our results highlight the lncRNA PIRAT (PU.1-induced regulator of alarmin transcription) as a regulator of exacerbated PU.1-dependent alarmin production during SARS-CoV-2 infection. A single nucleotide polymorphism (SNP) in the PIRAT locus has been associated with hematological malignancies (19); the function of PIRAT, however, has remained unknown. We characterize PIRAT as a nuclear RNA primarily expressed in CD14^+^ monocytes. PIRAT recruits the PU.1 transcription factor to pseudogenes and suppresses PU.1-binding to the S100A8 and S100A9 alarmin promoters. NF-κB–triggered down-regulation of PIRAT in monocytes upon PAMP stimulation or during severe COVID-19 consequently removes a transcriptional break on alarmin production. PIRAT down-regulation is accompanied by the up-regulation of the lncRNA LUCAT1 in monocytes, which propels alarmin induction in an NF-κB–dependent manner at the expense of the JAK-STAT pathway. Up-regulation of LUCAT1 and down-regulation of PIRAT thus alters PU.1, NF-κB, and JAK-STAT–dependent gene-expression in favor of the production of mediators associated with severe COVID-19.

## Results

### Identification of COVID-19 Relevant Myeloid lincRNA Signatures.

**Fig. 1.**
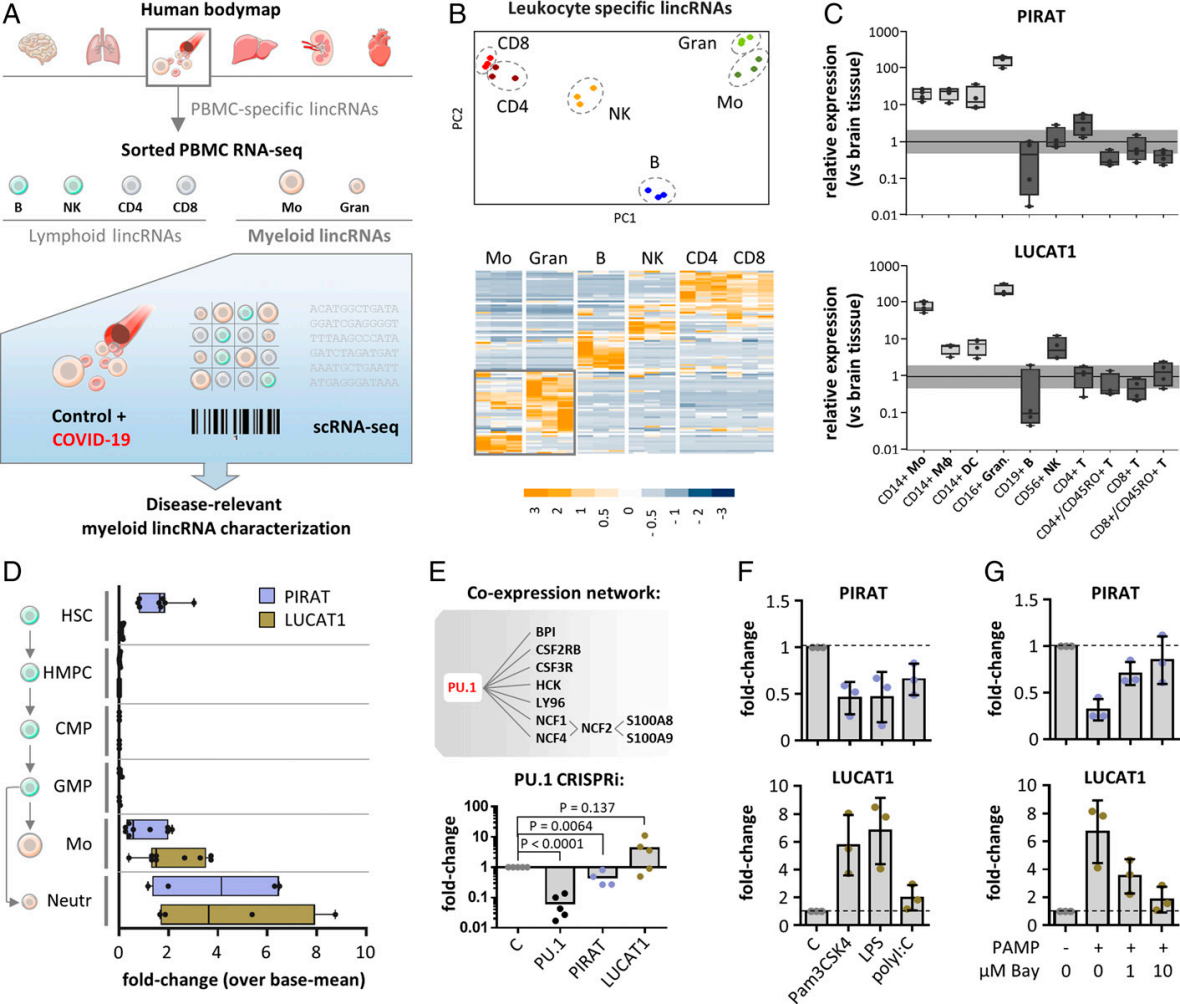
Identification of human myeloid lineage-specific lincRNAs. (*A*) Bulk and scRNA-seq strategy for the determination of myeloid lincRNAs relevant to COVID-19. (*B*) PCA (*Upper*) and hierarchical clustering (*Lower*, *z*-scores) of monocyte, granulocyte, B, NK, and CD4^+^ and CD8^+^ T cell lincRNA profiles. (*C*) qRT-PCR validation of PIRAT (LINC00211) and LUCAT1 as myeloid-specific lincRNAs (expression relative to human brain tissue). Horizontal bar indicates base-line (black) and twofold deviation from base-line (gray). (*D*) Relative abundance of PIRAT and LUCAT1 in RNA-seq profiles of hematopoietic stem cells (HSC), hematopoietic multipotent precursor cells (HMPC), common myeloid progenitors (CMP), granulocyte-macrophage progenitors (GMP), monocytes, and neutrophils. (*E*, *Upper*) PIRAT coexpression network, derived from RNA-seq data in *B*; (*Lower*) qRT-PCR analysis of PU.1 mRNA, PIRAT and LUCAT1 expression in PU.1 knockdown compared to control THP1 monocytes. (*F* and *G*) qRT-PCR analysis of lincRNA expression in response to indicated PAMPs and NF-κB inhibitor BAY-11-7082 (PAMP = 4 h LPS + polyI:C stimulation). (*C–G*) ≥3 independent experiments and one-way ANOVA test.

To chart candidate long intergenic noncoding RNAs (lincRNAs) relevant to disturbed myeloid immunity in COVID-19, we consolidated RNA-seq data from several sources, followed by in-depth scRNA-seq profiling (Fig. 1*A*). At first, leukocyte-specific mRNAs and lincRNAs were narrowed down using Illumina Human Bodymap data (Fig. 1*A*). Confirming successful extraction of leukocyte-specific RNAs from these datasets, pathway analysis revealed an exclusive enrichment of immune-relevant terms, such as “hematopoietic cell lineage,” “cytokine–cytokine receptor interaction,” or “chemokine signaling pathway” (*SI Appendix*, Fig. S1 *A* and *B*). We then charted expression of these transcripts among three publicly available replicates of peripheral blood monocyte, granulocyte, B cell, natural killer (NK) cell, and T cell RNA-seq profiles (20, 21). Principal component analysis (PCA) and hierarchical clustering successfully discriminated the major leukocyte compartments, based on their lincRNA and mRNA profiles, respectively (Fig. 1*B* and *SI Appendix*, Fig. S1 *C* and *D*). To confirm the cell-type specificity of the interrogated myeloid and lymphoid lincRNAs (*SI Appendix*, Table S1), we studied their expression in blood-derived macrophages, dendritic cells, monocytes, granulocytes, NK cells, B cells, and naïve (CD45RO^−^) or memory (CD45RO^+^) T cells. qRT-PCR confirmed preferential expression of LINC00211 (henceforth PIRAT), LUCAT1, and AC064805.1 in myeloid cells, whereas LINC02295, LINC02446, and LINC00861 were confirmed as lymphoid transcripts (Fig. 1*C* and *SI Appendix*, Fig. S1 *E–I*). Among the lymphoid lincRNAs, LINC02446 was particularly abundant in CD8^+^/CD45RO^+^ T cells, indicating a specific role in the CD8-memory niche (*SI Appendix*, Fig. S1 *H* and *I*). Among the myeloid lincRNAs, our attention was caught by PIRAT, since a SNP in the PIRAT locus (rs4670221-G, *P* value 3 × 10^−10^) had been associated with hematological alterations (19). The function of PIRAT, however, has remained unknown. Besides PIRAT, LUCAT1 was selected as a candidate lncRNA relevant to myeloid immunity in COVID-19 due to its particularly high expression in monocytes and granulocytes

To determine at which stages of myeloid ontogeny both lincRNAs become relevant, we traced their expression from hematopoietic stem cells (HSCs) to mature leukocytes, using Blueprint RNA-seq profiles (22). Expression of PIRAT declined upon HSC differentiation into multipotent progenitors and, similar to LUCAT1, remained low during the common myeloid and granulocyte/monocyte progenitor stages (Fig. 1*D*). Expression of both lincRNAs strongly increased in mature monocytes and neutrophils (Fig. 1*D*). Coexpression analysis using RNA-seq data from Fig. 1*B* suggested PIRAT to depend on a network driven by the myeloid master transcription factor PU.1 (Fig. 1*E* and *SI Appendix*, Fig. S2 *A* and *B*). Among the PIRAT-coexpressed genes were the PU.1-dependent alarmins S100A8 and S100A9 (Fig. 1*E* and *SI Appendix*, Fig. S2*B*), which play a key role in COVID-19 (5–7, 23, 24). Dependence of PIRAT but not LUCAT1 on PU.1 was confirmed by PU.1 knock-down in THP1 monocytes (Fig. 1*E*). Further underscoring their differential dependence on myeloid expression programs, PIRAT was down- and LUCAT1 was up-regulated in an NF-κB–dependent manner upon monocyte immune-activation (Fig. 1 *F* and *G* and *SI Appendix*, Fig. S2*C*). Thus, PIRAT and LUCAT1 are myeloid signature lncRNAs, activated during late hematopoiesis and differentially depending on PU.1 and NF-κB.

### Single-Cell Resolved Myeloid lincRNA Responses to SARS-CoV-2 Infection.

**Fig. 2.**
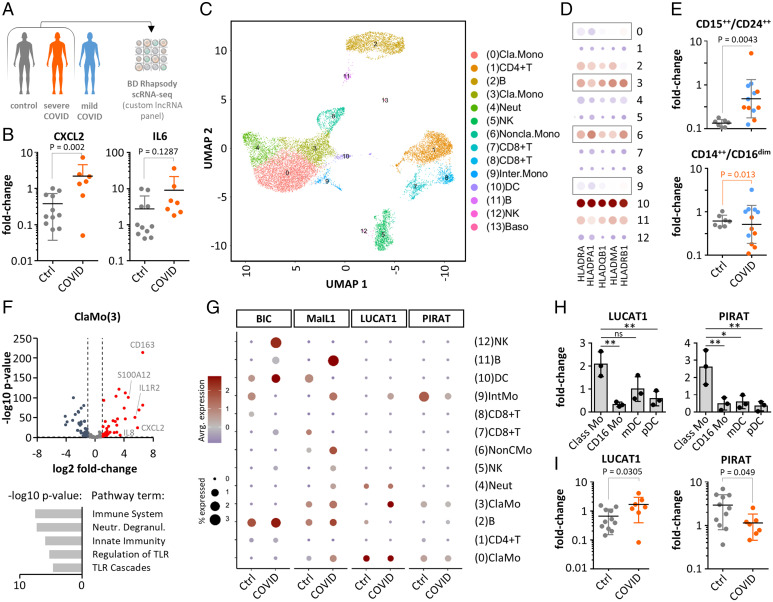
scRNA-seq analysis of lincRNA expression during COVID-19. (*A*) Patient PBMC analysis strategy. (*B*) Validation of immune marker induction in COVID-19 cohort PBMCs (qRT-PCR, control-patient 1 set as reference). (*C*) UMAP-plot with color-coded cell populations identified in merged scRNA-seq data. (*D*) HLA mRNA expression profile (scRNA-seq, monocytes highlighted). (*E*) FACS validation of immature neutrophil (CD15^++^/CD24^++^) appearance and reduction of classic monocytes (CD14^++^/CD16^dim^) in COVID-19 (color-coded according to *A*). (*F*) Volcano plot (*Upper*) and Reactome pathway (*Lower*) analysis of classic monocyte response during COVID-19 (scRNA-seq). (*G*) LincRNA profiles in control and COVID-19 patients (scRNA-seq). (*H*) PIRAT and LUCAT1 expression in classic (Class) and nonclassic (CD16) monocytes, myeloid dendritic cells (mDC), and plasmacytoid DCs (pDC) (qRT-PCR). (*I*) Same as *B*, but for PIRAT and LUCAT1. (*B*, *E*, and *I*) Two-tailed Student’s *t* test. (*H*) One-way ANOVA, three independent experiments. *P ≤ 0.05; **P ≤ 0.01.

Recent scRNA-seq studies have revealed profound changes in myeloid coding gene-expression in severe COVID-19. To dissect the contributions of myeloid lncRNAs, such as PIRAT and LUCAT1 to these alterations, we performed BD Rhapsody scRNA-seq of PBMCs from control and severe COVID-19 patients (World Health Organization [WHO] grade > 4) using an immune-response panel combined with a custom lncRNA panel (Fig. 2*A*) (patients listed in *SI Appendix*, Table S2). For qRT-PCR–based validation, we included PBMC samples from a second cohort without WHO grades available (*SI Appendix*, Table S3). qRT-PCR confirmed the expected induction of immune-response markers CXCL2 and IL-6 in COVID-19 patients from this cohort (Fig. 2*B*). scRNA-seq analysis of PBMCs from two control and two COVID-19 patients (WHO-graded cohort) (*SI Appendix*, Table S2) charted all expected myeloid and lymphoid populations and discriminated four monocyte populations along the CD14-, CD16-, and HLA-expression scheme (Fig. 2 *C* and *D* and *SI Appendix*, Fig. S3). FACS confirmed the reported increase in immature CD15^++^/CD24^++^ neutrophils and the reduction of CD14^++^/CD16^dim^ classic monocytes during severe COVID-19 (WHO grade > 4), indicative of myeloid exhaustion (5) (Fig. 2*E*; *SI Appendix*, Table S2). Differential gene expression and Reactome pathway analysis confirmed the proinflammatory activation of classic, nonclassic, and intermediate monocytes during COVID-19 (Fig. 2*F* and *SI Appendix*, Fig. S4).

Analysis of lincRNA scRNA-seq profiles confirmed the abundance of B cell proliferation promoting lncRNA BIC (25) in B lymphocytes. Furthermore, BIC was up-regulated in dendritic cells during COVID-19, in line with its role in antigen-presenting cell activation (Fig. 2*G* and *SI Appendix*, Fig. S5*A*) (26). Moreover, we observed the expected induction of type I IFN-inducing lincRNA MaIL1 (14) in all monocyte populations, but also in B cells from infected patients (Fig. 2*G* and *SI Appendix*, Fig. S5*B*). scRNA-seq also confirmed the strict myeloid expression of LUCAT1 and PIRAT and suggested preferential expression in CD14^+^-monocytes (Fig. 2*G* and *SI Appendix*, Fig. S5 *C* and *D*). Similarly, S100A8 and A9, which are coexpressed with PIRAT (Fig. 1*E*), were particularly highly expressed in myeloid cells (*SI Appendix*, Figs. S3*D* and S5 *E* and *F*). Unlike in classic and intermediate monocytes, LUCAT1 and PIRAT expression remained low in nonclassic CD16^+^-monocytes (Fig. 2*G*). Whereas LUCAT1 expression was up-regulated in classic and intermediate monocytes during COVID-19, PIRAT was down-regulated, reminiscent of the differential regulation of both lincRNAs in response to immune agonists (Fig. 2*G* compared to Fig. 1 *F* and *G*). Preferential expression of both lincRNAs in classic monocytes and opposite regulation during COVID-19 was confirmed in qRT-PCR experiments (Fig. 2 *H* and *I*). These results confirm an imbalanced myeloid compartment during severe COVID-19 and reveal LUCAT1 and PIRAT as CD14^+^ monocyte-specific lincRNAs, up- and down-regulated upon SARS-CoV-2 infection, respectively.

### LUCAT1 Attenuates STAT-Target Expression in Favor of Proinflammatory Genes in COVID-19.

**Fig. 3.**
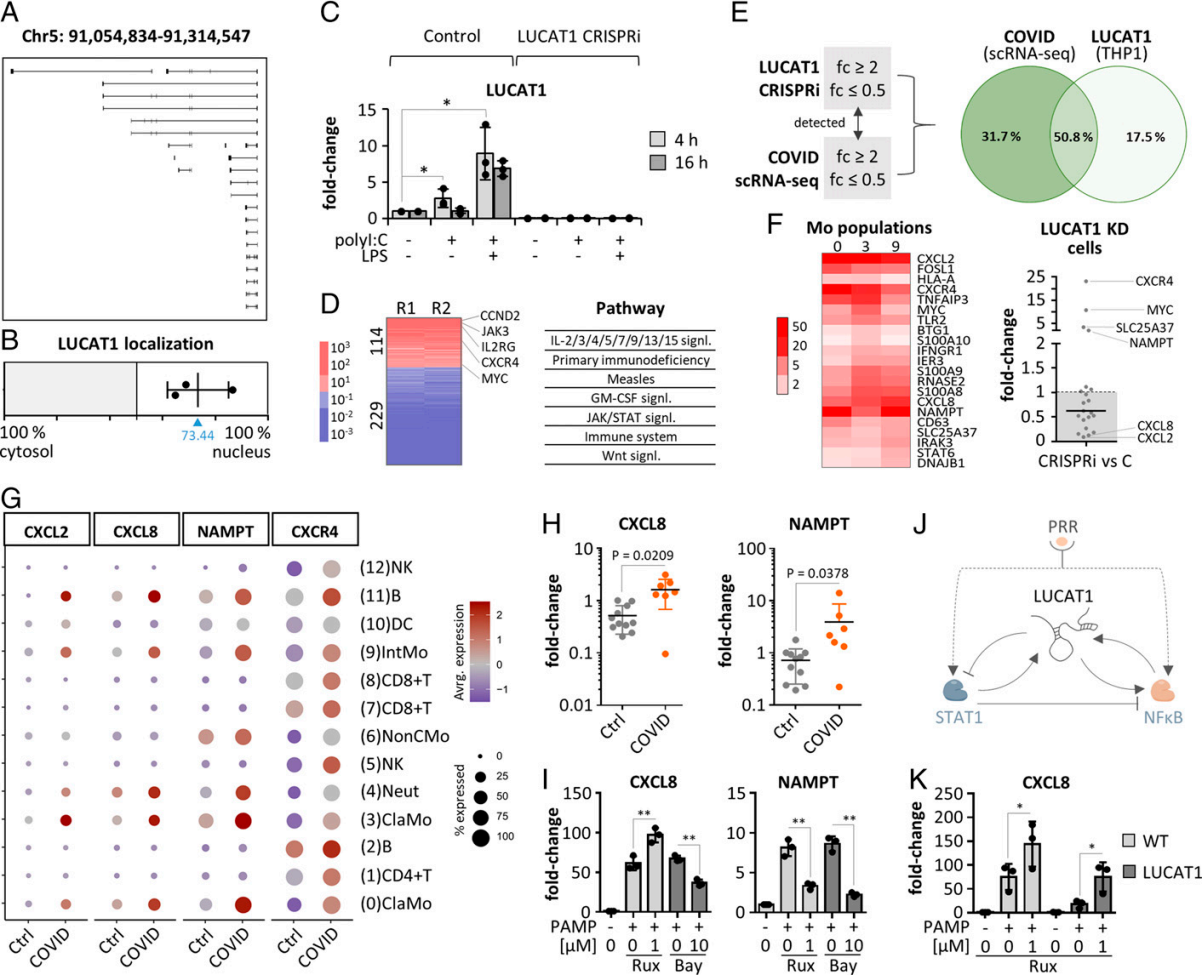
Role of LUCAT1 in monocytes. (*A*) ENSEMBL-annotated LUCAT1 isoforms. (*B*) LUCAT1 subcellular localization (qRT-PCR). (*C*) LUCAT1 expression in control and knockdown THP1 monocytes (qRT-PCR, relative to unstimulated control). (*D*) RNA-seq analysis (transcripts regulated ≥10-fold, Kyoto Encyclopedia of Genes and Genomes/Reactome pathways) of LUCAT1 knockdown versus control THP1 monocytes, activated for 4 h with polyI:C and LPS. R1 and R2 = replicates 1 and 2. (*E*) Overlap of gene regulations twofold or greater (up or down) in monocytes during COVID-19 (scRNA-seq populations 0, 3, and 9) (Fig. 2*C*) and upon LUCAT1 knockdown in THP1 cells. (*F*, *Left*) COVID-19-induced (twofold or greater) mRNAs in monocyte populations from Fig. 2*C*. (*Right*) Regulation of the same mRNAs in dataset from *D*. (*G*) Expression of LUCAT1-controlled mRNAs in scRNA-seq data. (*H*) Same as Fig. 2*B*, but for CXCL8 and NAMPT. (*I*) Ruxolitinib and BAY-11-7082 sensitivity of selected mRNAs (monocytes; PAMP = 4 h LPS + polyI:C). Fold-changes relative to unstimulated control. (*J*) Model of LUCAT1 function. (*K*) Rescue of CXCL8 dysregulation in LUCAT1-deficient THP1 cells upon 2-h Ruxolitinib pretreatment. (*C*, *I*, and *K*) One-way ANOVA, three independent experiments. (*H*) Two-tailed Student’s *t* test. *P ≤ 0.05; **P ≤ 0.01.

While our manuscript was in preparation, LUCAT1 was reported to act as a negative feedback regulator of JAK-STAT–dependent IFN immunity (27). LUCAT1 is a massively alternatively spliced lincRNA encoded on chromosome 5 (Fig. 3*A*) (27). Subcellular fractionation and qRT-PCR, based on the first and most frequently used exon, indicated a primarily nuclear localization in CD14^+^ monocytes (Fig. 3*B* and *SI Appendix*, Fig. S6*A*). To study its role in COVID-19, we silenced LUCAT1 in THP1 monocytes using CRISPR-interference (CRISPRi), followed by RNA-seq analysis and compared the results to patient scRNA-seq data. In line with our primary cell data (Fig. 1*F*), LUCAT1 expression increased in THP1 cells upon 4- or 16-h treatment with viral RNA analog polyI:C and bacterial LPS. In LUCAT1-CRISPRi cells, LUCAT1 expression was blunted under all conditions (Fig. 3*C*). Since LUCAT1 up-regulation was most pronounced after 4-h double-stimulation with polyI:C and LPS, this broad immune-activatory condition was selected for RNA-seq analysis. 114 mRNAs were up- and 229 were down-regulated ≥10-fold in PAMP-activated LUCAT1-deficient compared to control cells (Fig. 3*D*).

In agreement with previous reports (27), pathways relating to JAK-STAT–dependent receptors (e.g., IL-9R, IL-15R, or IL-2R) were enriched upon LUCAT1 knockdown (Fig. 3*D* and *SI Appendix*, Fig. S6*B*). mRNAs down-regulated upon LUCAT1 knockdown were associated with proinflammatory pathways, such as “TLR-signaling,” “chemokine receptor,” or “NF-κB signaling” (*SI Appendix*, Fig. S6*C*). To investigate the relevance of LUCAT1 in the context of SARS-CoV-2 infections, we compared mRNAs regulated twofold or greater (up or down) upon LUCAT1 knockdown in THP1 cells with mRNAs regulated twofold or greater (up or down) in classic and intermediate monocytes during COVID-19 (scRNA-seq data). Both datasets were reduced to mRNAs detected in both the THP1 CRISPRi and the scRNA-seq experiments (Fig. 3*E*); 50.8% of the mRNAs regulated in monocytes during COVID-19 were affected by LUCAT1 silencing (Fig. 3*E*). These mRNAs were associated with pathway terms, such as “rheumatoid arthritis,” “immune system,” or “NF-κB signaling” (*SI Appendix*, Fig. S6 *D* and *E*), indicating a broad influence of LUCAT1 on peripheral immunity during infection. When restricting the analysis to mRNAs up-regulated twofold or greater in monocytes during COVID-19 (Figs. 2*B* and 3 *F*–*H* and *SI Appendix*, Fig. S6*F*), the same dichotomy as in Fig. 3*D* was observed, with LUCAT1 deficiency lifting the expression of STAT-downstream genes (e.g., CXCR4 and NAMPT) and reducing classic proinflammatory marker expression (e.g., CXCL2 and CXCL8) (Fig. 3*F* and *SI Appendix*, Fig. S7*A*).

These results were confirmed in a second LUCAT1-CRISPRi cell line, using an independent guide RNA (gRNA) design (*SI Appendix*, Fig. S7*A*). BAY-11-7082 and Ruxolitinib inhibitor experiments confirmed the dependence of LUCAT1-controled proinflammatory markers CXCL2 and CXCL8 on the NF-κB but not the JAK-STAT pathway, whereas CXCR4 and NAMPT were JAK-STAT–dependent (Fig. 3*I* and *SI Appendix*, Fig. S7*B*). LUCAT1 itself was found to depend both on the NF-κB and the JAK-STAT pathway (*SI Appendix*, Fig. S7*B* and Fig. 1*G*). Thus, LUCAT1 up-regulation during monocyte activation in COVID-19 likely restrains JAK-STAT signaling, in favor of NF-κB–dependent immunity. Interestingly, treatment of monocytes with the STAT-inhibitor and COVID-19 drug (28) Ruxolitinib not only reduced the expression of STAT-targets CXCR4 and NAMPT, but also increased the expression of proinflammatory markers CXCL8 and CXCL2 (Fig. 3*I* and *SI Appendix*, Fig. S7*B*). This suggests that STAT inhibition by LUCAT1 not only restrains STAT-target expression but also eliminates a STAT-dependent break on NF-κB target genes (Fig. 3*J*). In line with this model, treatment of LUCAT1-deficient cells with Ruxolitinib restored CXCL8 and CXCL2 expression and reverted the overexpression of STAT targets CXCR4 and NAMPT (Fig. 3*K* and *SI Appendix*, Fig. S7*C*). Thus, LUCAT1 likely links negative feedback control of the JAK-STAT axis to NFκB target gene expression in PAMP-challenged monocytes (Fig. 3*J*).

### COVID-Suppressed lincRNA PIRAT Antagonizes Alarmin Expression in Monocytes.

**Fig. 4.**
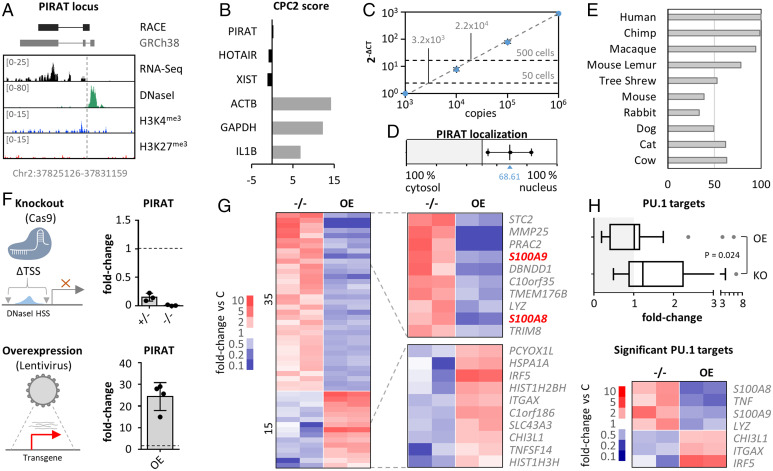
Role of PIRAT in human monocytes. (*A*) RACE-PCR refined (black) and annotated (gray) PIRAT splice structure and chromosomal position, compared to ENCODE primary CD14^+^-monocyte RNA-seq, DNaseI-seq, and ChIP-seq (H3K4me3 and H3K27me3) coverage. Track-height indicated in brackets. (*B*) CPC2 coding score of indicated lncRNAs and mRNAs. (*C*) PIRAT copy number enumeration by absolute qPCR, relative to PIRAT RNA standard. Two independent analysis (each three independent replicates), using RNA worth 50 and 500 CD14^+^ monocytes, respectively. Average PIRAT copy number (not yet divided by the number on input cells) is shown . (*D*) Subcellular localization of PIRAT in primary CD14^+^-monocytes (qRT-PCR, three independent experiments; C = cytoplasm, N = nucleus). (*E*) Conservation of RACE-PCR refined PIRAT sequence in the respective species (percentages). (*F*) Representation and qRT-PCR-validation of PIRAT mono- (^+/−^) and biallelic (^−/−^) knockout and lentiviral overexpression (OE) strategy (THP1 monocytes). (*G*) RNA-seq analysis of PIRAT knockout (^−/−^) and overexpression (OE) cells (color-coded mRNA fold-changes ≥ 2, compared to wild-type cells). (*H*, *Upper*) Base-mean fold-changes of PU.1 target genes in datasets from *G*. (*Lower*) PU.1-controled genes, significantly regulated (twofold or greater, *P* ≤ 0.05) into opposite directions after PIRAT knockout and overexpression, respectively. (*H*) Two-tailed Student’s *t* test.

We next deciphered the function of the uncharacterized lincRNA PIRAT in human monocytes and the reasons for its opposite regulation compared to LUCAT1 in COVID-19. First, we mapped the exact PIRAT architecture by RACE-PCR. Deviating from the GENCODE annotation, 5′ and 3′ RACE revealed a two-exon structure in primary monocytes (Fig. 4*A* and *SI Appendix*, Fig. S8). ENCODE monocyte RNA-seq, DNaseI-seq and chromatin immunoprecipitation-sequencing (ChIP-seq) data confirmed a DNaseI hypersensitive site at the mapped PIRAT 5′-end and H3K4 trimethylation and RNA-seq coverage across the RACE-refined gene body, hallmarks of transcriptionally active regions (Fig. 4*A*). The CPC2 algorithm confirmed low coding potential of the refined PIRAT sequence, similar to the noncoding RNAs XIST and HOTAIR, and different from mRNAs (ACTB, GAPDH, IL1B) (Fig. 4*B*). Copy-number enumeration by absolute quantification qRT-PCR indicated ∼40 to 60 PIRAT copies per primary CD14^+^ monocyte (Fig. 4*C* and *SI Appendix*, Fig. S9 *A–E*), similar to other functional lncRNAs (14, 29). Subcellular fractionation characterized PIRAT as a nuclear-retained lincRNA (Fig. 4*D*), which was further corroborated by RNA-FISH (*SI Appendix*, Fig. S9*F*). PIRAT sequence conservation exceeded 90% in the genomes of catarrhine primates but dropped to 33.5% in mice (Fig. 4*E* and *SI Appendix*, Fig. S9*G*). Thus, PIRAT is a two-exon nuclear lincRNA, stably maintained during higher primate evolution.

To study the function of PIRAT, we generated PIRAT promoter-deficient THP1 cells using CRISPR/Cas9 (Fig. 4*F* and *SI Appendix*, Fig. S9*H*) and cells overexpressing PIRAT from a lentiviral backbone (Fig. 4*F*). RNA-seq uncovered dozens of mRNAs regulated (twofold or greater) into opposite directions upon PIRAT knockout and overexpression, respectively (Fig. 4*G*). Among the top 10 PIRAT-suppressed genes were the PU.1-dependent alarmins S100A8 and S100A9 (Fig. 4*G*). S100A8 and A9 form a heterodimer, referred to as calprotectin, which plays important roles in the immune system, ranging from promyelopoietic to immunomodulatory and metabolic functions, relevant to a wide range of diseases (30), including COVID-19 (5, 7, 23, 24). S100A8 and S100A9 are coexpressed with PIRAT at the PBMC whole-population level (Fig.1*E*) but negatively correlate with PIRAT expression at the single-cell level (*SI Appendix*, Fig. S10*A*). This further hints at a role of PIRAT as an intrinsic negative regulator of a PU.1-driven module, driving S100A8, S100A9, and PIRAT expression in myeloid cells. Beyond S100A8/A9, the suppressive effect of PIRAT extended to other PU.1-driven genes (Fig. 4*H* and *SI Appendix*, Fig. S10*B* and Table S4). Reciprocally, genes suppressed by PU.1, such as ITGAX (CD11c) or CHI3L1 (31–33), were derepressed upon PIRAT knockout (Fig. 4*H*). Thus, PIRAT is a myeloid nuclear RNA, restraining the expression of PU.1-driven genes, such as S100A8 and S100A9.

**Fig. 5.**
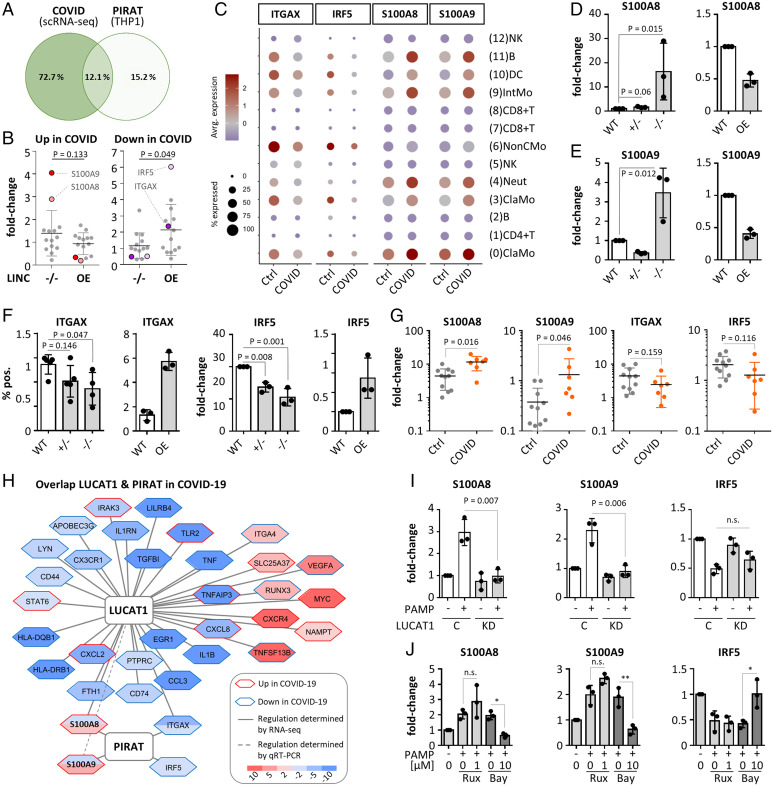
Participation of PIRAT in PU.1 circuits relevant to COVID-19. (*A*) Overlap of genes regulated twofold or greater in classic monocytes during COVID-19 (scRNA-seq data) and upon PIRAT knockout/overexpression (data from Fig. 4*G*). (*B*) Regulation of genes in PIRAT knockout (^−/−^) or overexpression (OE) compared to wild-type cells, up-regulated (*Left*) or down-regulated (*Right*) during COVID-19. (*C*) Cell-type–specific expression of top PIRAT-controlled, COVID-responsive mRNAs from *B* (scRNA-seq). (*D*–*F*) Expression changes of PIRAT-controlled PU.1 targets in PIRAT knockout and overexpression compared to wild-type THP1 cells (qRT-PCR). (*G*) Regulation of PIRAT-controlled genes in PBMCs from COVID-19 and control-patients (qRT-PCR, control-patient 1 set as reference). (*H*) Overlap of LUCAT1 and PIRAT controlled genes (THP1 RNA-seq data from *A* and Fig. 3*E*), regulated twofold or greater (up or down). Fill-colors indicate regulations due to lncRNA deficiency. Plot reduced to genes regulated twofold or greater (up or down) during COVID-19 (scRNA-seq, Fig. 2). (*I*) Regulation of S100A8, S100A9, and IRF5 in control and LUCAT1-CRISPRi cells from Fig. 3 (4 h LPS and polyI:C). (*J*) Same as Fig. 3*I*, but for S100A8, S100A9, and IRF5. (*D*–*F* and *I* and *J*) One-way ANOVA; (*G*) two-tailed Student’s *t* test; ≥3 independent experiments. *P ≤ 0.05; **P ≤ 0.01.

Next, we overlaid the RNA-seq profiles of PIRAT-manipulated cell lines with the scRNA-seq profiles of COVID-19 and control patient PBMCs. Among all mRNAs up- or down-regulated twofold or greater during COVID-19 (scRNA-seq data, classic monocytes) or upon PIRAT expression-manipulation (THP1 monocytes), 33 were detected in both datasets. The overlap of mRNAs regulated twofold or greater in both datasets was 12.1% (four mRNAs) (Fig. 5*A* and *SI Appendix*, Fig. S11*A*), and these mRNAs fell into immune-relevant categories, such as “Toll-like receptor cascades” (*SI Appendix*, Fig. S11*B*). Similarly, COVID- and PIRAT-specific regulations, respectively, were associated with immune- and infection-specific terms (*SI Appendix*, Fig. S11 *C* and *D*). Among the mRNAs up-regulated during COVID-19 (scRNA-seq data), S100A8 and S100A9 experienced the strongest derepression upon PIRAT knockout (Fig. 5 *B* and *C*). Vice versa, genes down-regulated in CD14^+^-monocytes during severe COVID-19 were under significant positive influence by PIRAT, headed by the PU.1-suppressed genes IRF5 and ITGAX (Fig. 5 *B* and *C*). ITGAX (CD11c) is a cell surface integrin of inflammatory monocytes, elevated in mild courses of COVID-19 (5). IRF5 is a transcription factor involved the production of type I IFN and other immune mediators and has been suggested as a therapy-relevant COVID-19 marker (34, 35). Thus, disease-relevant genes activated and suppressed by PIRAT are reciprocally regulated by PU.1 and in COVID-19. This notion was further corroborated in qRT-PCR and FACS validations, which confirmed the control of S100A8, S100A9, ITGAX, and IRF5 by PIRAT and regulation of these factors during COVID-19 (Fig. 5 *D*–*F*, and *G* and *SI Appendix*, Fig. S11 *E* and *F*). Finally, knockdown of PU.1 in THP1 monocytes using CRISPR interference verified the dependence not only of PIRAT, but also of S100A8 and S100A9 on this transcription factor (Fig. 1*E* and *SI Appendix*, Fig. S11*G*). These data suggest PIRAT as a negative feedback regulator of PU.1, limiting S100A8 and A9 alarmin expression in monocytes at base-line. NF-κB–dependent down-regulation of PIRAT (Fig. 1*G*) consequently removes a molecular break on the production of alarmins.

To determine the reason for the opposite regulation of PIRAT and LUCAT1 during COVID-19, we compared the influence of both lincRNAs on genes regulated in CD14^+^ monocytes in patients. Comparison of all mRNAs regulated twofold or greater (up or down) during COVID-19 in CD14^+^ monocytes and upon silencing of either lincRNA in THP1 cells (overlaps from Venn diagrams in Figs. 3*E* and 5*A*), suggested only a small overlap in the regulatory networks of PIRAT and LUCAT1 (Fig. 5*H*). In line with our assumptions (Figs. 3 *H*–*K* and ), ENRICHR transcription factor analysis predicted COVID-relevant genes up- and down-regulated upon LUCAT1-loss to depend on STAT and NF-κB (RelA), respectively, whereas PIRAT-controlled genes were predicted to depend on IRF8 (rank 1) and PU.1 (= SPI1, rank 2) (*SI Appendix*, Fig. S12*A*). Among the few mRNAs influenced by both lincRNAs was S100A8, which is up-regulated upon loss of PIRAT in naïve cells, and down-regulated upon LUCAT1 silencing in PAMP-challenged cells (Fig. 5 *H* and *I*). S100A9 was down-regulated in only one RNA-seq replicate after LUCAT1 silencing (0.509- and 1.614-fold); qRT-PCR, however, confirmed a significant reduction of S100A9 expression in LUCAT1-deficient cells, similar to S100A8 (Fig. 5*I* and *SI Appendix*, Fig. S12*B*). Up-regulation of both alarmins upon PAMP-stimulation was NF-κB–dependent (Fig. 5*J*), in line with the elimination of STAT-dependent NF-κB target suppression by LUCAT1 (Fig. 3) and in line with the NF-κB–dependent down-regulation of PIRAT during monocyte activation (Fig. 1*G*).

Of note, STAT-inhibition in LUCAT1-deficient cells partially restored S100A8 and A9 expression (*SI Appendix*, Fig. S12*C*). IRF5, a PIRAT target, predicted by our RNA-seq data not to be influenced by LUCAT1, was confirmed to remain unaffected by LUCAT1-silencing or JAK-STAT inhibition (Fig. 5 *H*–*J*). Taken together, our results suggest PIRAT and LUCAT1 to regulate largely discrete sets of genes in CD14^+^ monocytes during COVID-19, with LUCAT1 inhibiting STAT and promoting NF-κB target gene expression and PIRAT serving as a withdrawable inhibitor of PU.1-dependent programs. S100A8 and S100A9 depend both on the NF-κB pathway, promoted by LUCAT1, and the PU.1-pathway, suppressed by PIRAT. As a result, the opposite regulation of both lincRNAs in COVID-19 likely supports the production of these critical alarmins.

### PIRAT Suppresses PU.1 Binding to Alarmin Promoters and Fosters Its Association with Pseudogenes.

**Fig. 6.**
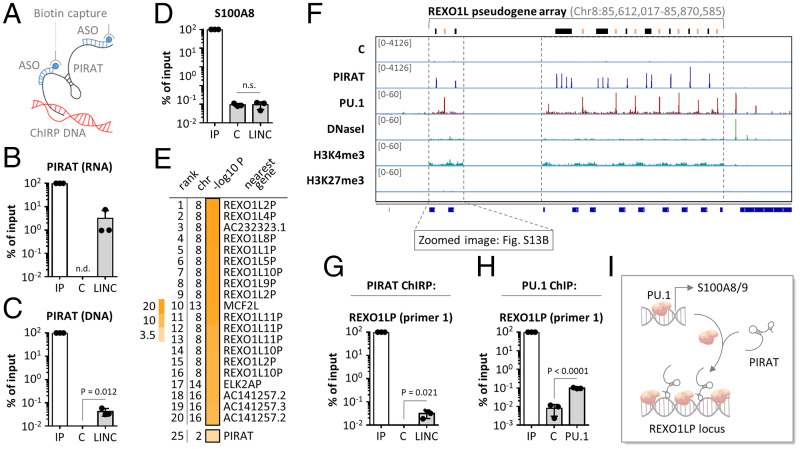
Repetitive binding of PU.1 and PIRAT to the REXO1LP locus. (*A*) PIRAT ChIRP was performed using primary CD14^+^ monocytes. (*B*) Recovery of PIRAT RNA in ChIRP samples compared to input (IP) sample (C = control ChIRP, LINC = PIRAT ChIRP; qRT-PCR). (*C*) Same as *B* but with genomic DNA. (*D*) Same as *C* but with S100A8 promoter detection. (*E*) Summary of PIRAT binding site peak-calling (ChIRP-seq; chr = chromosome; top 20 peaks and peak #25 are shown; full list of peaks in *SI Appendix*, Table S5). (*F*) IGV plots showing control (C) and PIRAT ChIRP-seq and matched CD14^+^-monocyte PU.1 ChIP-, DNaseI-, and histone-3 ChIP-seq coverage in the REXO1LP locus. Track-heightin brackets. (*G*) qRT-PCR validation of PIRAT binding to ChIRP peaks in the REXO1LP locus. (*H*) qRT-PCR validation of PU.1 binding to ChIP peaks in the REXO1LP locus. (*I*) Model of PU.1 redirection from alarmin promoters to the REXO1LP locus by PIRAT. (*B*–*D*, *G*, and *H*) Two-tailed Student’s *t* test, three independent experiments.

To interrogate the molecular mechanism of alarmin control by PIRAT, we investigated the interaction of this lincRNA with chromatin and PU.1. Antisense-purification of PIRAT-occupied chromatin from primary monocytes by chromatin isolation by RNA purification (ChIRP) (Fig. 6*A*) recovered PIRAT RNA and verified cross-linking of PIRAT to its own site of transcription (Fig. 6 *B* and *C*). Refusing a model where the lincRNA controls PU.1 directly at its target gene promoters, PIRAT did not bind to the PU.1 occupied region upstream of the S100A8 gene (Fig. 6*D* and *SI Appendix*, Fig. S13*A*). In search of alternative explanations, we recorded the genome occupancy profile of PIRAT in CD14^+^-monocytes using ChIRP-seq. Peak-calling, comparing PIRAT ChIRP-seq signals to a control ChIRP-seq library, revealed PIRAT to occupy multiple sites along the uncharacterized REXO1L-pseudogene array at chromosome 8q21.2 (Fig. 6*E* and *SI Appendix*, Table S5). Comparison to matched ENCODE CD14^+^ monocyte ChIP-seq data uncovered a repetitive pattern of alternating PIRAT and PU.1 binding sites along the entire open chromatin of the REXO1LP repeat (Fig. 6*F* and *SI Appendix*, Fig. S13*B*). The identified PIRAT occupied sequences in this locus only differ at single nucleotide positions (*SI Appendix*, Fig. S14*A*). The same is true for the PU.1 sites in this locus (*SI Appendix*, Fig. S14*B*). ChIRP– and ChIP–qRT-PCR, using alternative primer pairs directed against the concatenated, REXO1LP-specific PIRAT- and PU.1-peak sequences confirmed PIRAT and PU.1 interaction with REXO1LP repeat DNA (Fig. 6 *G* and *H* and *SI Appendix*, Fig. S15*A*).

**Fig. 7.**
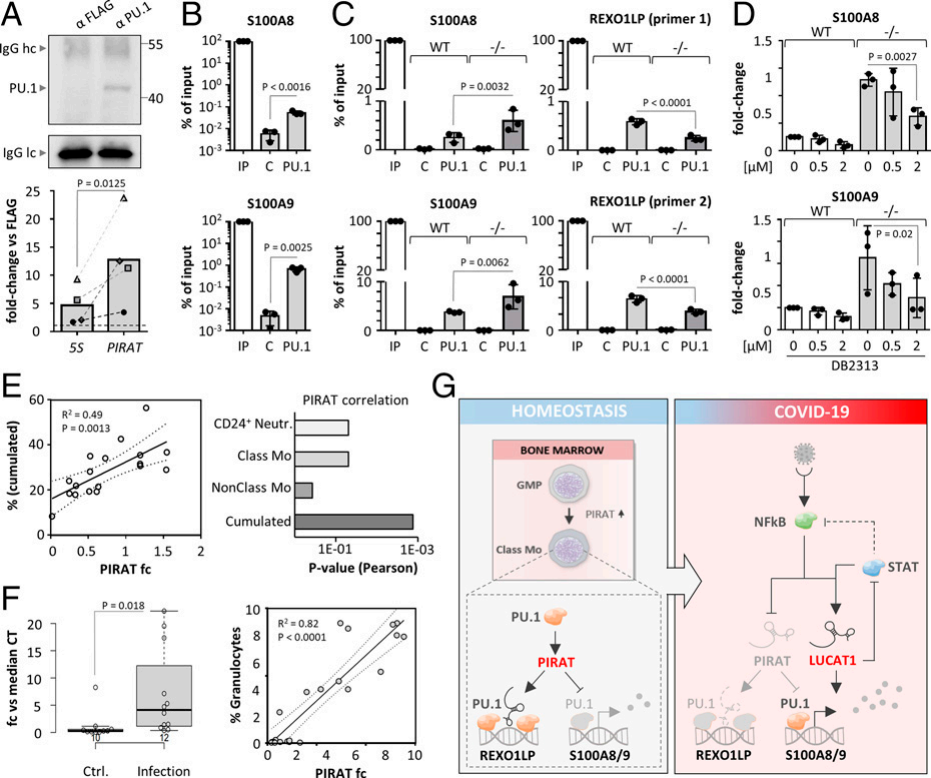
PIRAT redirects PU.1 from alarmin promoters to the REXO1LP repeat. (*A*) Western blot validation of PU.1 capture (*Upper*; hc/lc = light chain/heavy chain) and qRT-PCR analysis of PIRAT enrichment (*Lower*) in PU.1 UV-CLIP (primary monocytes). (*B*) ChIP qRT-PCR analysis of PU.1 binding to S100A8 and A9 promoter DNA in primary monocytes. (*C*) qRT-PCR analysis of PU.1 binding to ChIP-seq peaks in the S100A8 and S1009 promoters and the REXO1LP locus, in wild-type (WT) and PIRAT knockout (^−/−^) THP1 cells. (*D*) qRT-PCR analysis of S100A8 and S100A9 expression after treatment of wild-type (WT) and PIRAT knockout (^−/−^) THP1 cells with PU.1 inhibitor DB2313 (concentrations indicated) for 4 h. (*E*, *Left*) Pearson correlation of cumulated neutrophil and classic monocyte percentage with PIRAT levels in COVID-19 patient PBMCs. (*Right*) *P* values for PIRAT correlation (Pearson) with percentages of the indicated cell types in COVID-19 patient PBMCs. (*F*, *Left*) qRT-PCR analysis of PIRAT expression in BALF from control and pulmonary infection patients. (*Right*) Pearson correlation of granulocyte percentage with PIRAT expression in BALF. (*G*) Summary of alarmin-control by PIRAT and LUCAT1 in monocytes. (*A*, *B*, and *F*) Two-tailed Student’s *t* test. (*C* and *D*) One-way ANOVA; ≥3 independent experiments were performed. Abbreviations: C, control; IP, input.

Subcloning of qRT-PCR products from PIRAT ChIRP eluates (*SI Appendix*, Fig. S15*A*), followed by Sanger sequencing, discriminated at least four PIRAT binding sites, differing at single nucleotide positions, respectively (*SI Appendix*, Fig. S15 *B* and *C*). These nucleotide variations are not annotated in the current GENCODE GRCh38 human reference genome, potentially, due to the difficulty of repeat sequence reconstructions (36). All obtained Sanger sequences exclusively mapped to the REXO1LP locus (allowing up to 10 mismatches) (*SI Appendix*, Fig. S15*D*), underscoring their origin from this locus. Notwithstanding possible uncertainties in REXO1LP locus annotation, these data support the possibility of PIRAT-mediated redirection of PU.1 from alarmin promoters to REXO1LP sites (Fig. 6*I*). In line with such a decoy function, PIRAT interacted with PU.1 in primary monocytes in UV-CLIP experiments (∼12-fold enrichment) (Fig. 7*A* and *SI Appendix*, Fig. S16*A*). ChIP confirmed PU.1 binding to the promoters of S100A8 and A9 in primary monocytes (Fig. 7*B* and *SI Appendix*, Fig. S13*A*), which was enhanced in PIRAT-deficient compared to wild-type THP1 monocytes (Fig. 7*C*). Concurrently, PU.1-binding to the repeated REXO1LP sites was diluted in the absence of PIRAT (Fig. 7*C*). This supports the hypothesis, that PIRAT dampens alarmin expression in naïve monocytes by redirecting PU.1 from alarmin promoters to pseudogene binding sites. To verify that the increase in alarmin expression upon PIRAT knockout is PU.1-dependent, we treated PIRAT-deficient cells with the small molecule DB2313, which inhibits chromatin-binding of PU.1 (37). PU.1 inhibitor treatment not only reduced PIRAT expression in wild-type THP1 cells, but also counteracted the increased S100A8 and S100A9 expression in PIRAT-deficient cells in a dose-dependent manner (Fig. 7*D* and *SI Appendix*, Fig. S16*B*). Thus, PIRAT inhibits alarmin expression as a negative feedback regulator of PU.1 in the nucleus of human monocytes.

### PIRAT Is a Myeloid Marker with Clinical Utility beyond COVID-19.

Given the specific myeloid expression and function of PIRAT, we predicted its utility as a marker of myeloid cell abundance and tissue infiltration in infectious and inflammatory diseases. Indeed, expression of PIRAT in PBMC samples from control and COVID-19 patients correlated with the relative abundance of CD24^+^ neutrophils and classic monocytes, but not with nonclassic monocytes (Fig. 7*E*), in agreement with little expression of PIRAT in the latter (Fig. 2). Beyond COVID-19, PIRAT levels correlated with the percentage of infiltrating myeloid cells (granulocytes; *R*^2^ = 0.82) in bronchoalveolar lavage fluid (BALF) from patients with bronchopulmonary infection (Fig. 7*F*). To test the utility of PIRAT as a myeloid infiltration marker in noninfectious lung diseases, we measured PIRAT in idiopathic pulmonary fibrosis (IPF) tissue. Neutrophils play an important role in IPF tissue remodeling and elevated migration of these cells into IPF tissue has been associated with early mortality (38). PIRAT levels significantly correlated with the percentage of neutrophils in IPF tissue (*R*^2^ = 0.83) but not with NK cells (*R*^2^ = 0.14) (*SI Appendix*, Fig. S16*C*). Thus, PIRAT is a suitable marker for myeloid cell abundance in patient biomaterial, in line with its important role in the myeloid system.

In summary, our results suggest a vital role of lincRNAs as regulators of immune mediator production in the myeloid lineage during COVID-19. Activation of PIRAT upon differentiation of myeloid precursors in the bone marrow likely establishes a break on PU.1-dependent S100A8 and A9 expression by redirecting PU.1 to pseudogenes. NF-κB–dependent down-regulation of PIRAT during infection enhances PU.1 binding to the S100A8 and A9 promoters. Simultaneously, the NF-κB and JAK-STAT pathways promote the expression of LUCAT1, which further propels the production of alarmins and classic NF-κB responsive factors, such as CXCL8, at the expense of STAT-dependent immunity. Collectively, PIRAT down-regulation and LUCAT1 up-regulation in monocytes in this model fuels the expression of S100A8 and S100A9, which contribute to myeloid imbalances during severe COVID-19 (Fig. 7*G*).

## Discussion

Besides characteristic cellular changes, indicative of emergency myelopoiesis, severe COVID-19 entails systemic inflammatory components also registered in other difficult to treat infectious disease trajectories (1, 2). A better understanding of the underlying molecular circuits is urgently needed to improve the outcome of infections with SARS-CoV-2 and other potentially pandemic agents. Several recent studies have employed scRNA-seq to dissect peripheral immune alterations in COVID-19 (3, 5, 7). So far, however, the noncoding RNA layer has been neglected.

Here, we employed an lncRNA-centric approach to dissect mechanisms underlying immune-alterations in COVID-19 at the single-cell level. Our results reveal the lincRNA PIRAT to be primarily expressed in monocytes, a critical source of peripheral immune-mediators, such as S100A8 and A9 in COVID-19 (7). We also find PIRAT to be expressed in granulocytes (Fig. 1*C*) and to correlate with granulocyte counts in biomaterial from diseased tissue (Fig. 7*F* and *SI Appendix*, Fig. S16*C*). As granulocytes are major sources of alarmins (6), expression of which is restrained by PIRAT, it would be worthwhile to investigate the role of PIRAT in this cell type. Upon their release from monocytes and granulocytes, S100A8 and S100A9 form the calprotectin complex, which has intricate pro- and antiinflammatory functions and influences cellular metabolism and cytoskeletal processes in various cell types (30, 39, 40). Furthermore, S100A8 and S100A9 expression may be uncoupled. S-nitrosylated S100A8 has, for example, been reported to suppress mast cell degranulation (30, 39, 41). In line with their pleiotropic functions, S100A8 and S100A9 have been implicated in a variety of diseases, ranging from arthritis to diabetes and cardiovascular diseases (30, 39, 40). Therefore, PIRAT, as a regulator of these alarmins, could be involved in other diseases beyond COVID-19.

Upstream of S100A8 and A9, PIRAT controls PU.1 as a negative feedback regulator. Feedback control constitutes a universal regulatory principle, conferring stability to cellular circuits (42). Mechanistically, PIRAT inhibits PU.1 association with alarmin promoters and fosters PU.1 binding to the REXO1LP locus, which suggests a novel function of pseudogenes as nuclear caches for transcription factors. An open question concerns how PU.1 recruitment to the REXO1LP locus by PIRAT is achieved mechanistically. Despite the alternating binding pattern, PIRAT- and PU.1-occupied regions in this locus do not overlap. The physical association of PIRAT with PU.1 (Fig. 7*A*) suggests that chromatin loops may occur, bringing PIRAT and PU.1 binding sites into spatial proximity at the REXO1LP locus. This could result in a condensed, PU.1-inhibiting chromatin focus that is maintained by PIRAT. Further experiments, for example using single-molecule RNA-FISH with PU.1 costaining or PU.1 ChIA-PET experiments, could further narrow down the mechanism of PIRAT-dependent PU.1 recruitment into this locus. Since PU.1 is a master-regulator of myelopoiesis, PIRAT might also contribute to the imbalanced myeloid differentiation trajectories seen in severe COVID-19, independent of S100A8 and A9. The PU.1 dose, for example, decides over the commitment to the macrophage and granulocyte differentiation paths, respectively (43, 44). Furthermore, reduction of PU.1 levels is required for megakaryocyte differentiation and thus platelet production (45). This might also explain the association of a SNP in the *PIRAT* locus with altered platelet volume (19). Granulocyte and platelet differentiation trajectories again are disturbed in COVID-19 (5, 46). In vivo studies could further clarify the role of PIRAT in myeloid cell differentiation and activation. The low sequence conservation of PIRAT in rodents (Fig. 4*E*), however, calls into question the possibility of such investigations.

Besides PIRAT, other lncRNAs have been reported to act in the myeloid niche. Schwarzer et al. (47) identified *LINC00173* as a regulator of myeloid progenitor proliferation, contributing to granulopoiesis, probably through PRC2 complex-dependent modifications at HOX-gene loci. Similarly, the lncRNA Hotairm1 was found to regulate granulocytic differentiation and HOX gene expression through a yet unknown mechanism (48, 49). During terminal myeloid differentiation, PU.1-induced lncRNA *lnc-MC* was reported to promote monocyte-to-macrophage differentiation (50). These seminal studies support the notion that lncRNAs critically contribute to the timing of myelopoietic programs and suggest that PIRAT is embedded into a larger regulatory RNA network in myeloid cells.

Due to their important roles in the immune system, lncRNAs such as PIRAT should be considered potential pharmacological targets. Recent successes in antisense-directed therapies (51) and antisense-manipulation of myeloid RNA-circuits (52) make PIRAT targeting therapeutics seem feasible. Further immune-regulatory lncRNAs, such as MaIL1, GAPLINC, PACER, or CARLR, could become relevant in this context as well. MaIL1, for example, supports type I IFN immunity, which in turn is counter-regulated by LUCAT1 (14, 27). Both lincRNAs are up-regulated during COVID-19 (Fig. 2*G*). IFN-STAT pathway inhibition by Ruxolitinib has been reported to prevent the progression of COVID-19 with systemic hyperinflammation into multiorgan-failure (28). Thus, whereas MaIL1 could nurture COVID-19 pathogenesis, LUCAT1 might adopt a protective function, preventing excessive IFN-STAT-driven immune responses. Importantly, however, LUCAT1 seems to contribute to the production of alarmins, associated with severe courses of COVID-19. This suggests that pharmacological intervention in lncRNA circuits needs to be considered with similar care as the use of conventional pathway inhibitors, such as Ruxolitinib.

In summary, our results suggest a multistaged model of immunoregulation in COVID-19 and other infectious diseases, in which lncRNAs occupy a central position. In the myeloid system, lncRNAs such as PIRAT and LUCAT1 control the activity of immune master-transcription factors such as PU.1 and STAT1 via complex feedback mechanisms. Negative feedback between PU.1 and PIRAT in resting cells ensures that downstream production of the critical alarmins S100A8/A9 is kept within narrow limits. Under inflammatory conditions, PIRAT-dependent alarmin suppression is lifted and alarmin production is further promoted by LUCAT1, which ties JAK/STAT inhibition to NF-κB–dependent gene expression. Correspondingly, malfunctions at the lincRNA level are anticipated to have a decisive influence on the transcription factor networks determining the course of COVID-19 and other immune-associated diseases.

## Materials and Methods

### Cell Culture and Human Biomaterial.

Buffy coats were obtained from the transfusion medicine department, University Hospital of Giessen and Marburg, Giessen, and deidentified prior to use. THP1 and Hek293T cells were obtained from ATCC. All cells were cultured at 37 °C in a humidified atmosphere with 5% CO_2_. Cell purification, culture and stimulation conditions are further specified in *SI Appendix*, *Supplementary Methods*.

COVID-19 patients (*SI Appendix*, Tables S2 and S3) were tested positive for SARS-CoV-2 RNA in nasopharyngeal swabs. The BioInflame study was approved by the ethics committee of the Charité-Universitätsmedizin Berlin (EA2/030/09) and the University Medical Center Marburg (55/17). BALF (Fig. 7*F*) was obtained at the University Clinics Giessen and Marburg (ethics aproval Marburg: 87/12) or at Charité, Berlin (ethics approval EA2/086/16). Late stage IPF tissue was obtained from the UGMLC Giessen Biobank/eurIPF registry biobank, member of the DZL Platform Biobanking, on approval by ethics committee (Az 58/15 and 111/08). Patient characteristics are listed in *SI Appendix*, Table S6. BAL procedure, study design, and patient characteristics are further detailed in *SI Appendix*, *Supplementary Methods*.

### Cell Manipulation.

For gene silencing the pX458 vector system (53) (*SI Appendix*, Fig. S9*H*) or a lentiviral CRISPR interference vector (54) was used (Addgene #71237). gRNA sequences are provided in *SI Appendix*, Table S7.

For PIRAT overexpression, the SparQ lentivector (Systembio, # QM511B-1) was used. Detailed procedures are provided in the *SI Appendix, Supplementary Methods*.

### PCR and Cloning.

DNA from PIRAT ChIRP elutions was amplifed using Advantage 2 polymerase (Takara) and subcloned using the Strataclone TA PCR cloning kit (Agilent), followed by Sanger sequencing (Seqlab GmbH).

RACE-PCR was performed using the SMARTer 5′/3′ RACE kit (Clontech) and products were subcloned and sequenced as above. For detailed procedures see *SI Appendix*, *Supplementary Methods*.

### Copy Number Enumeration.

PIRAT copy number was determined by qRT-PCR reative to a synthesized PIRAT RNA standard as described in *SI Appendix*, *Supplementary Methods*.

### Subcellular Fractionation.

Cytoplasm and nucleus were separated by differential centrifugation, followed by RNA extraction, as detailed in *SI Appendix, Supplementary Methods*.

### Nucleic Acid and Protein Detection.

For RNA and DNA detection by quantitative PCR, the High-Capacity cDNA Reverse Transcription Kit and PowerUP SYBR Green Master Mix (Thermo Fisher) or the Power SYBR RNA-to-Ct 1-Step Kit (Thermo Fisher) was used. Expression changes were calculated using the 2−ΔΔCT method. RNA-FISH was performed using the ViewRNATM ISH Tissue 1-Plex Assay (Affymetrix) (14). For Western Blot, 10% polyacrylamide SDS PAGE gels, nitrocellulose membranes and a Chemostar Imager (INTAS Science Imaging) were used. For details, see *SI Appendix*, *Supplementary Methods*.

### Flow Cytometry.

Cells were stained with fluorophore-coupled antibodies and analysed using a Guava EasyCyte (Millipore) instrument. For details, see *SI Appendix, Supplementary Methods*.

### Chromatin and Protein Affinity Purification.

ChIP was performed by coupling magnetic beads to PU.1 C1 + A7 antibody or FLAG antibody (*SI Appendix*, Table S8), as described by Tawk et al. (55). *SI Appendix*, *Supplementary Methods*.

ChIRP was performed using 3′ monobiotinylated antisense DNA probes (*SI Appendix*, Table S9) as described previously (56).

For co-IP, the procedure published by Tawk et al. (55) was used with minor modifications using antibodies listed in *SI Appendix*, Table S8. For details, see *SI Appendix*, *Supplementary Methods*.

### Single-Cell RNA-Sequencing Analysis.

Single-cell multiomics was performed using the BD Rhapsody system and the Human Immune Response Panel supplemented with custom-made primers for additional genes. For details see *SI Appendix*, *Supplementary Methods*.

### Bulk Sequencing and Bioinformatics Analysis.

Illumina TruSeq mRNA libraries and Cross-linking immunoprecipitiation (CLIP)-seq and ChIRP-seq libraries (Vertis Biotech AG) were sequenced on a HiSeq 1500 or a NexSeq500 machine. Further sequencing data were obtained through public sequence read archives (see *SI Appendix, Supplementary Methods*).

For RNA-seq and ChIP-seq data analysis and visualization tools and strategies see *SI Appendix*, *Supplementary Methods*.

### Statistical Analysis.

Statistical analysis was performed based on at least three independent experiments, except for scRNA-seq experiments. Test details can be found in the figure legends and methods details. If not specified differently, GraphPad Prism software was used for two-tailed Student’s *t* test and ANOVA analysis. Differences between two or more compared conditions were regarded significant when *P* values were ≤ 0.05. Where possible, *P* values are shown in the respective figure panels.

## Supplementary Material

Supplementary File

## Data Availability

Bulk and scRNA-seq data have been deposited in the NCBI GEO database, https://www.ncbi.nlm.nih.gov/geo (accession no. GSE142503) (58) and are publicly available as of the date of publication.
