# Supplementary material for: Single-cell RNA sequencing uncovers the nuclear decoy lincRNA PIRAT as a regulator of systemic monocyte immunity during COVID-19
Source: Proc Natl Acad Sci U S A. 2022 Aug 23;119(36):e2120680119. doi: 10.1073/pnas.2120680119 (PMC9457492; doi:10.1073/pnas.2120680119)
Supplement: Supplementary File [file pnas.2120680119.sapp.pdf]

Supplementary Information for

Single cell RNA sequencing uncovers the nuclear decoy lincRNA PIRAT as a regulator of systemic monocyte immunity during COVID-19.

Marina Aznaourova†, Nils Schmerer†, Harshavardhan Janga, Zhenhua Zhang, Kim Pauck, Judith Bushe, Sarah M Volkers, Daniel Wendisch, Philipp Georg, Evgenia Ntini, Michelle Aillaud, Margrit Gündisch, Elisabeth Mack, Chrysanthi Skevaki, Christian Keller, Christian Bauer, Wilhelm Bertrams, Annalisa Marsico, Andrea Nist, Thorsten Stiewe, Achim D Gruber, Clemens Ruppert, Yang Li, Holger Garn, Leif E Sander, Bernd Schmeck and Leon N Schulte*.

*Leon N Schulte
Email: leon.schulte@uni-marburg.de

This PDF file includes:

Figures S1 to S16
Tables S1 to S9
Supplementary Methods

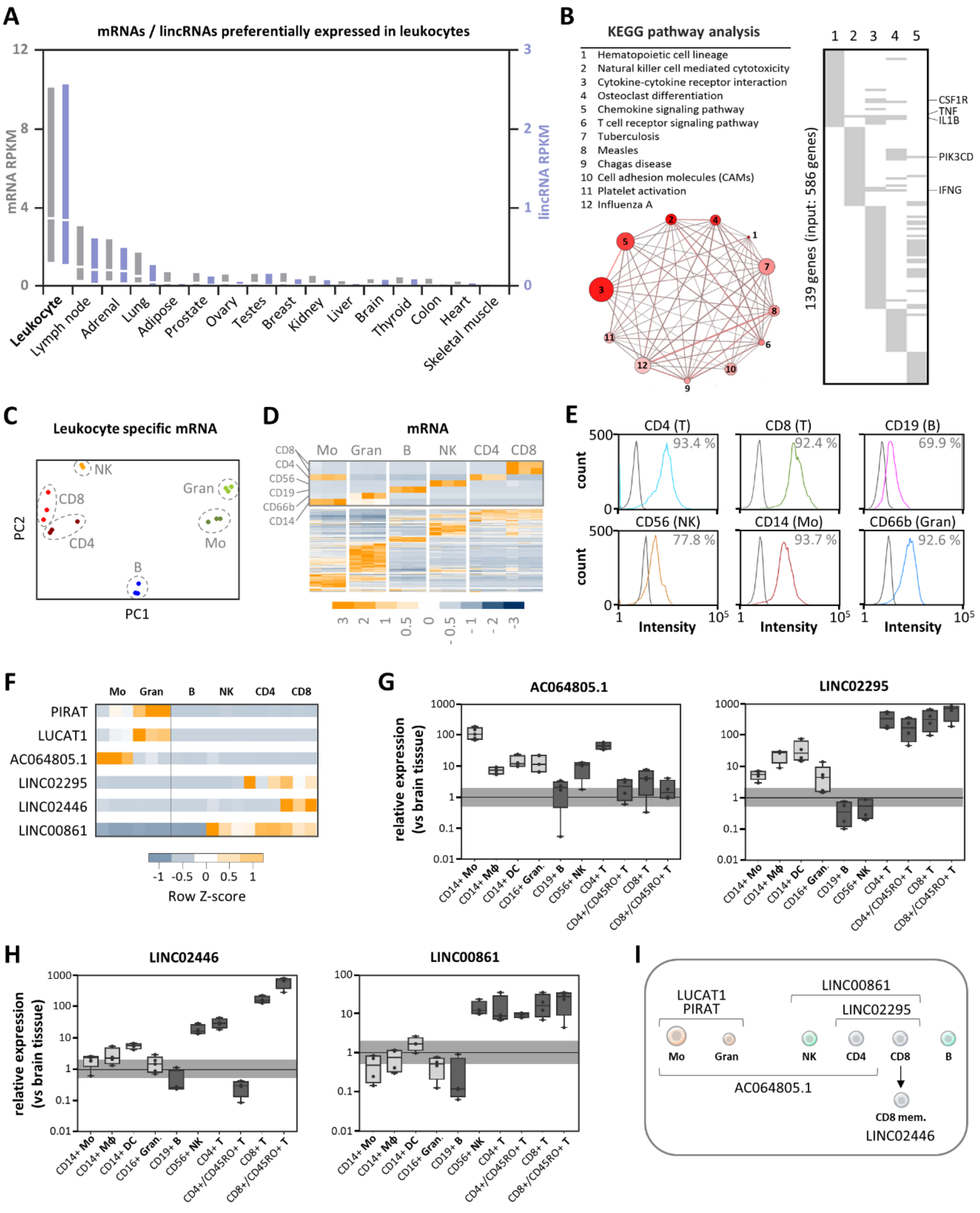

Fig. S1. Identification of myeloid and lymphoid lincRNAs signatures. A) mRNA and lincRNA expression levels (median + inner quartiles) in indicated tissues (Human Bodymap). B) Consensus Path DB KEGG pathway analysis (top left) and induced network analysis (lower left) of mRNAs from A. Pathway size = circle size. Candidates contained: color-coded (light to dark red). Overlap between pathways = line thickness. Overlap of genes contained in the top 5 pathways is illustrated in the right panel (each grey cell stands for one gene). Genes annotated in at least 3 signaling pathways are indicated. C) and D) PCA analysis with leukocyte-enriched lincRNAs and clustering of mRNAs from A (cell type markers indicated). E) Overlay histogram FACS plots showing

successful enrichment of the indicated cell populations by MACS (left peak: unstained control; right peak: cells stained for indicated surface antigen). F) RNA-Seq based row Z-scores of selected myeloid (top 3) and lymphoid (bottom 3) lincRNA markers (data from Fig. 1B). G) and H) qRT-PCR validation of lincRNAs from indicated purified cell types, relative to human brain reference tissue. Horizontal bar indicates base-line (black) and 2-fold deviation from base-line (grey). Box plots and individual replicate values from four independent experiments are shown. I) Summary of lincRNA expression patterns in the studied leukocyte populations.

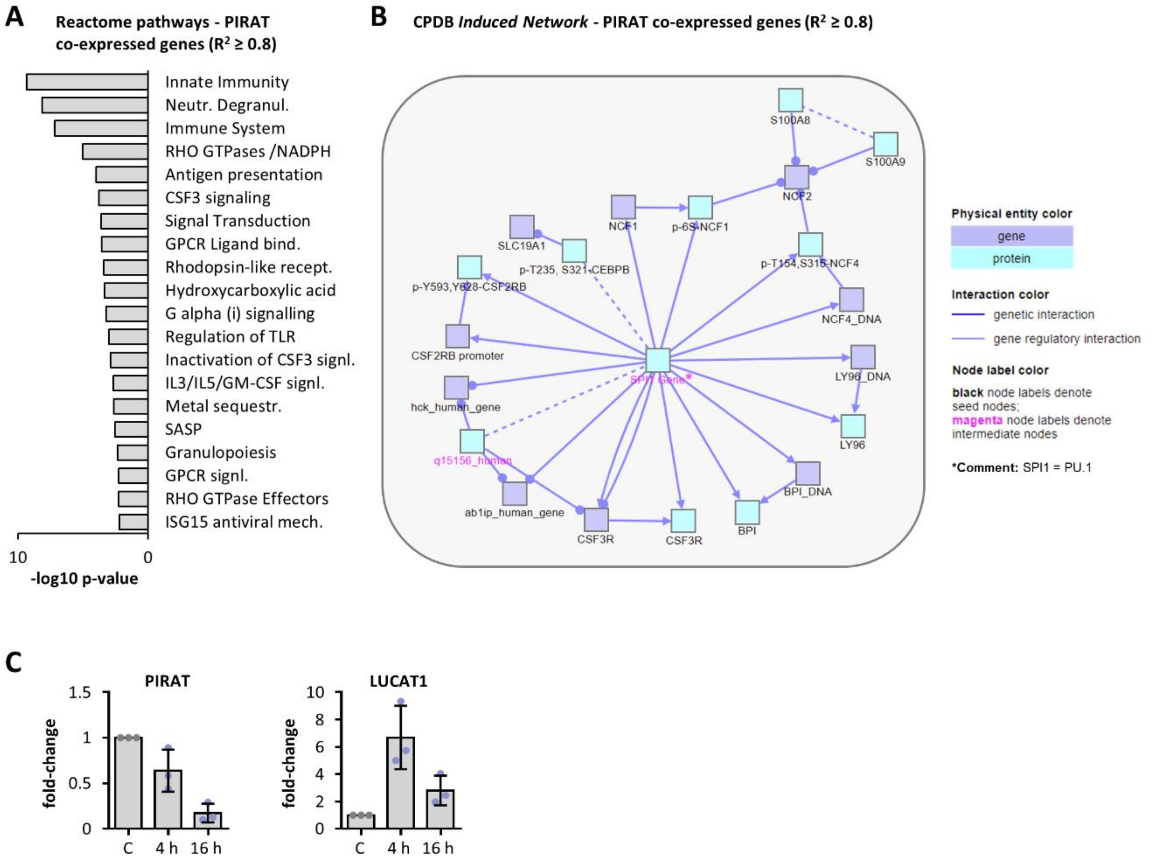

Fig. S2. PIRAT co-expression network. A) Consensus Path DB reactome pathway analysis and B) Consensus Path DB induced network analysis of genes co-expressed with PIRAT in RNA-seq datasets from Fig. 1. C) qRT-PCR analysis of PIRAT and LUCAT1 expression in primary monocytes in response to LPS + polyI:C (4 and 16 h stimulation, compared to respective unstimulated control). C: 3 independent experiments.

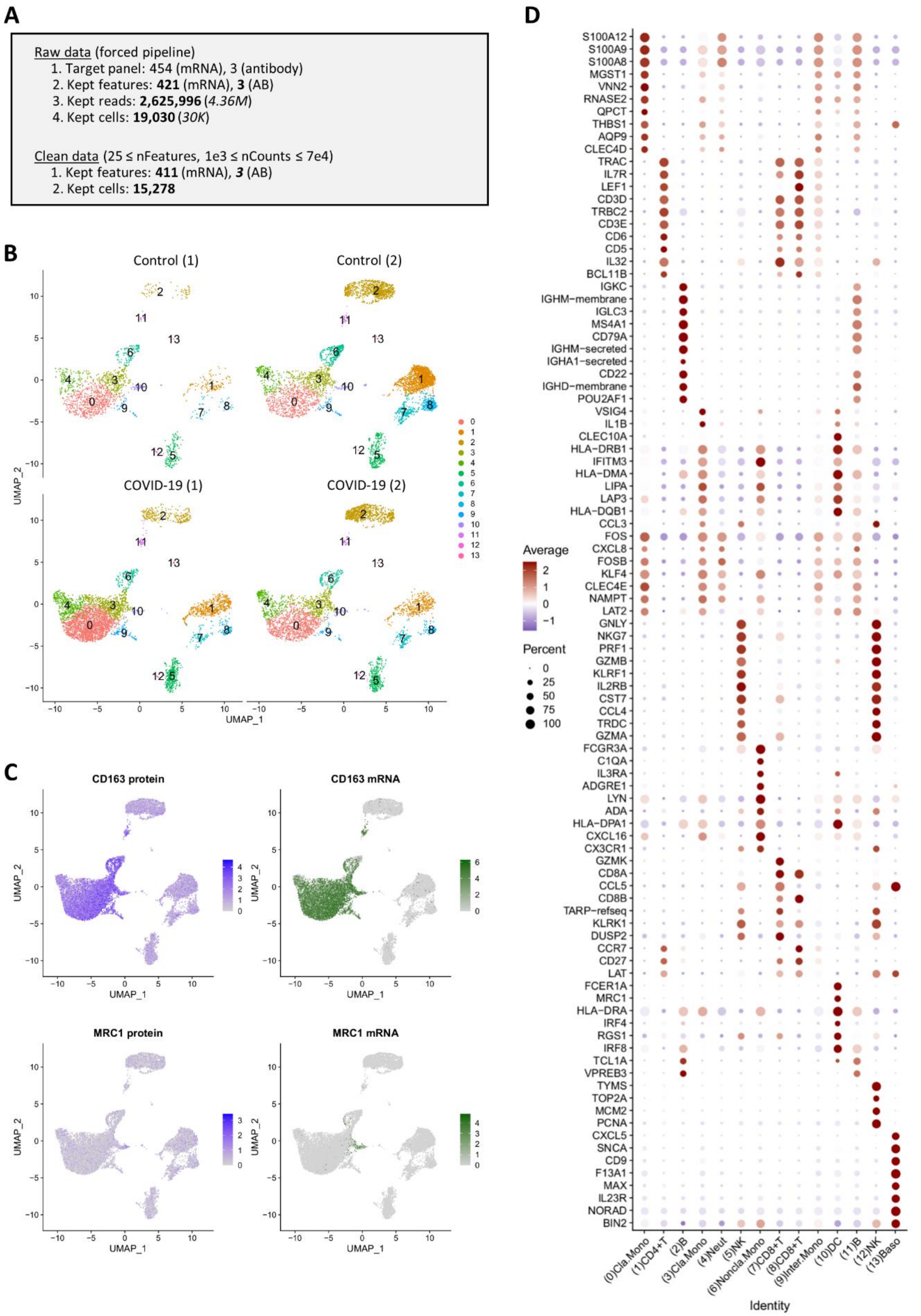

A
Raw data (forced pipeline)
1. Target panel: 454 (mRNA), 3 (antibody)
2. Kept features: 421 (mRNA), 3 (AB)
3. Kept reads: 2,625,996 (4.36M)
4. Kept cells: 19,030 (30K)
Clean data (25 ≤ nFeatures, 1e3 ≤ nCounts ≤ 7e4)
1. Kept features: 411 (mRNA), 3 (AB)
2. Kept cells: 15,278
B
Control (1)
Control (2)
COVID-19 (1)
COVID-19 (2)
UMAP_1
UMAP_2
C
CD163 protein
CD163 mRNA
MRC1 protein
MRC1 mRNA
D
Average
Percent
(0)Cla.Mono
(1)CD4+T
(2)B
(3)Cla.Mono
(4)Neut
(5)NK
(6)Noncla.Mono
(7)CD8+T
(8)CD8+T
(9)Inter.Mono
(10)DC
(11)B
(12)NK
(13)Baso
Identity

Fig. S3. PBMC single cell RNA-seq data analysis. A) Illustration of data analysis strategy and statistics. B) UMAP plots showing the clustering of cell types in the indicated control and COVID-19 patient scRNA-seq datasets. C) UMAP plots showing surface protein marker (AbSeq) and mRNA detection for CD163 (myeloid marker) and MRC1 (dendritic cell / monocyte marker) in aggregated control and COVID-19 patient scRNA-seq data. D) Dot plot showing the color-coded average expression and the percentage of positive cells for characteristic mRNA/lincRNA markers in the indicated cell populations.

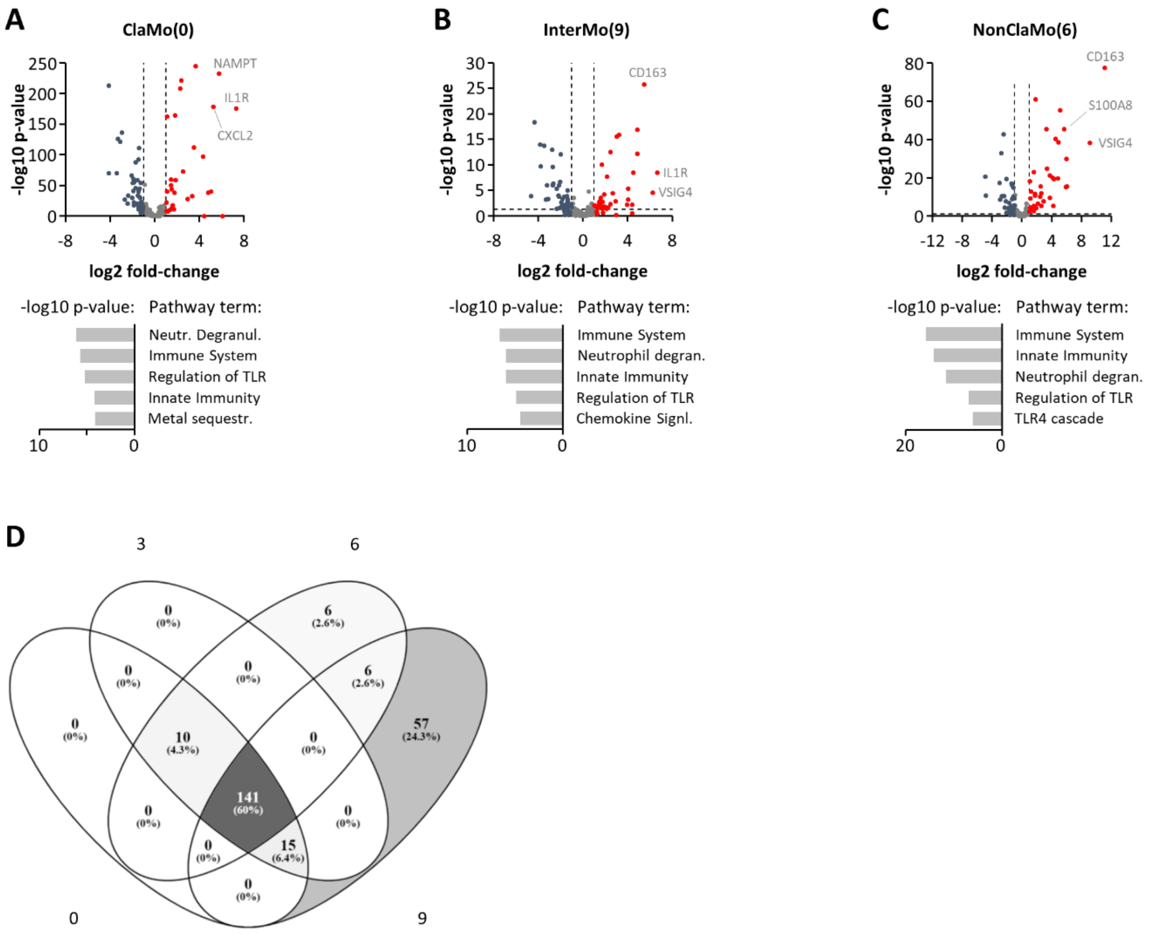

Fig. S4. Monocyte response in COVID-19 (differential gene expression). A) to C) Top: volcano plots showing gene expression changes in classical, intermediate and non-classical monocyte populations in COVID-19 compared to control patients (based on scRNA-seq data shown in Fig. S3). Top 3 induced mRNAs are indicated. Bottom: Consensus Path DB Reactome pathway analysis with significantly up-regulated mRNAs (top 5 pathways are shown). D) Overlap of differentially expressed genes in the respective monocyte populations (numbers denote cell populations defined in Fig. 2C and Fig. S3D).

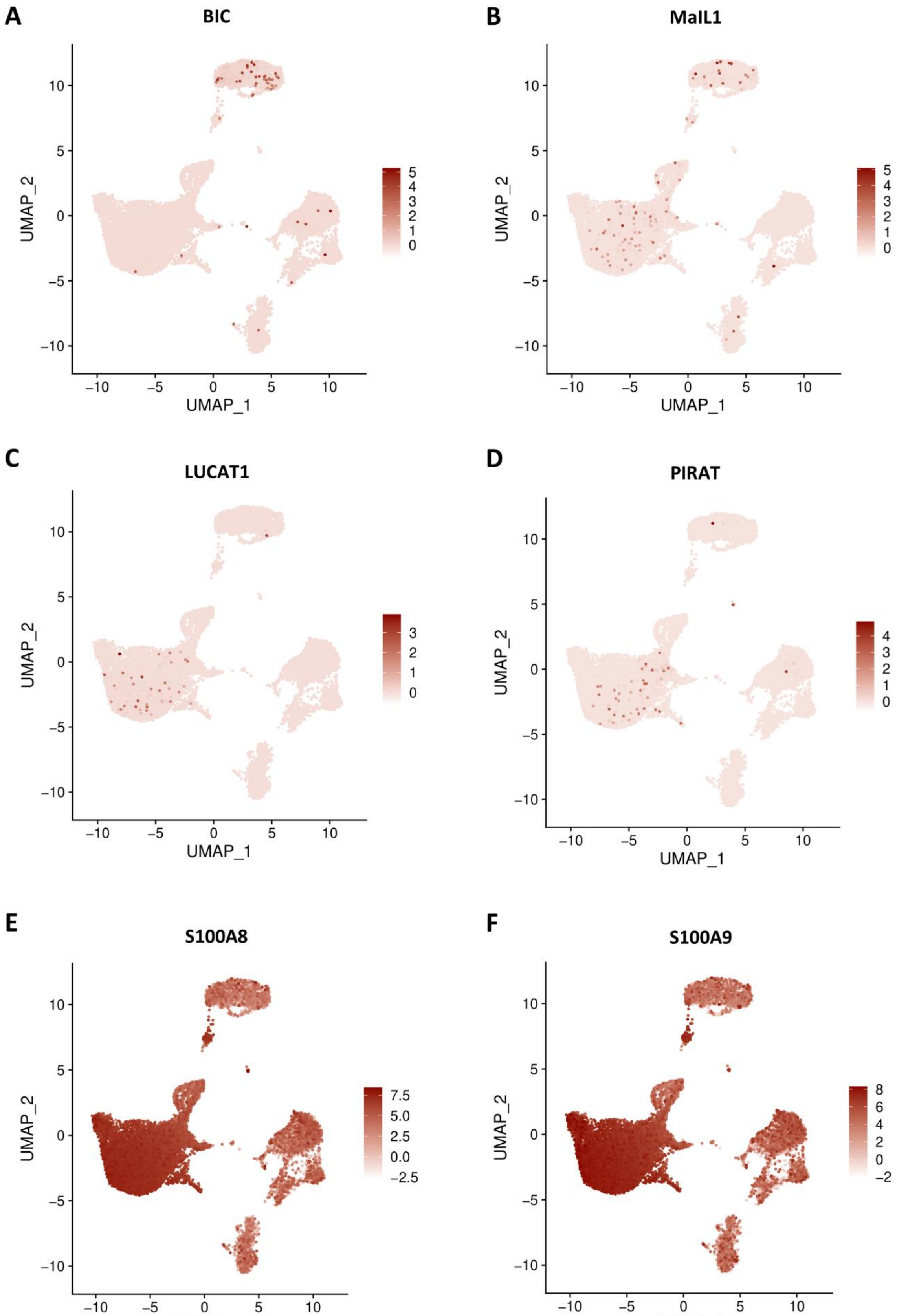
A
BIC
B
MaIL1
C
LUCAT1
D
PIRAT
E
S100A8
F
S100A9
UMAP_1
UMAP_2

Fig. S5. UMAP analysis of marker expression in scRNA-seq data. A-F) UMAP plots illustrating the distribution of BIC, MaIL1, LUCAT1, PIRAT, S100A8 and S100A9 positive cells in scRNA-seq data from Fig. 2. Background: single cell populations from Fig. 2C. Red dots: Cells positive for the respective marker (expression level color-coded according to the legend in each plot; [min-max scale]).

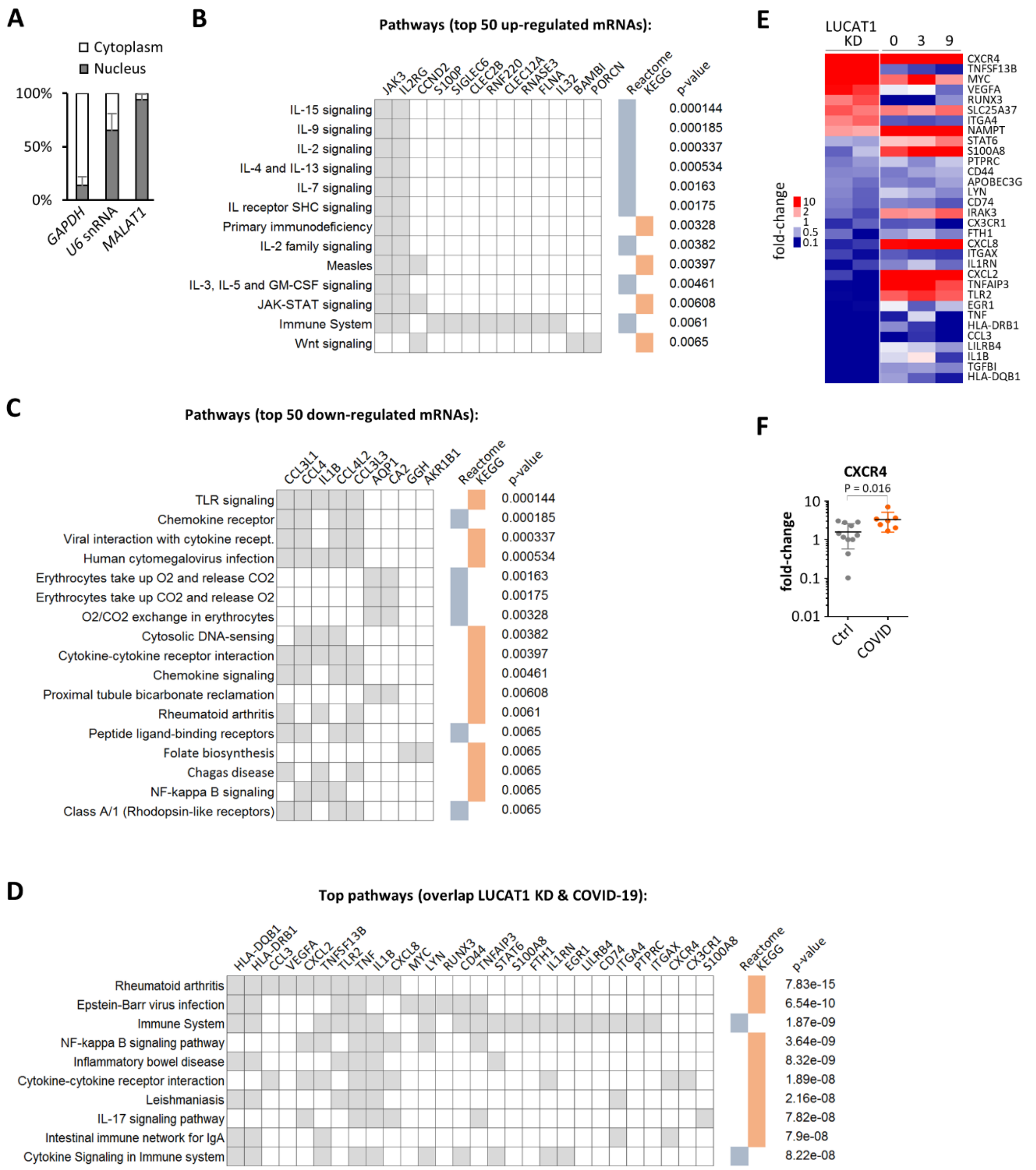

Fig. S6. Influence of LUCAT1 on the human monocyte immune response. A) Primary human monocyte subcellular fractionation qRT-PCR. Distribution of GAPDH mRNA, U6 snRNA and the MALAT1 lncRNA in nuclear and cytoplasmic fractions is shown in percent (mean and standard-deviation, based on three independent experiments). B-C) Consensus Path DB KEGG and Reactome pathway analysis of top 50 genes, up-regulated (panel B) or down-regulated (panel C) upon LUCAT1 knockdown. Pathway terms, pertaining genes, pathway sources and p-values are shown. Grey fill color indicates that a given gene is included in the respective pathway. D) Same as B-C, but with genes from Fig. 3E (Venn diagram overlap). E) Heatmap, showing the fold-changes of genes regulated ≥ 2-fold (up or down) upon LUCAT1 knockdown (THP1 RNA-seq data) and in the indicated monocyte populations in COVID-19 compared to control patients (scRNA-seq data). F) Validation of CXCR4 up-regulation in COVID-19 cohort whole PBMCs (qRT-PCR, control-patient 1 set as reference).

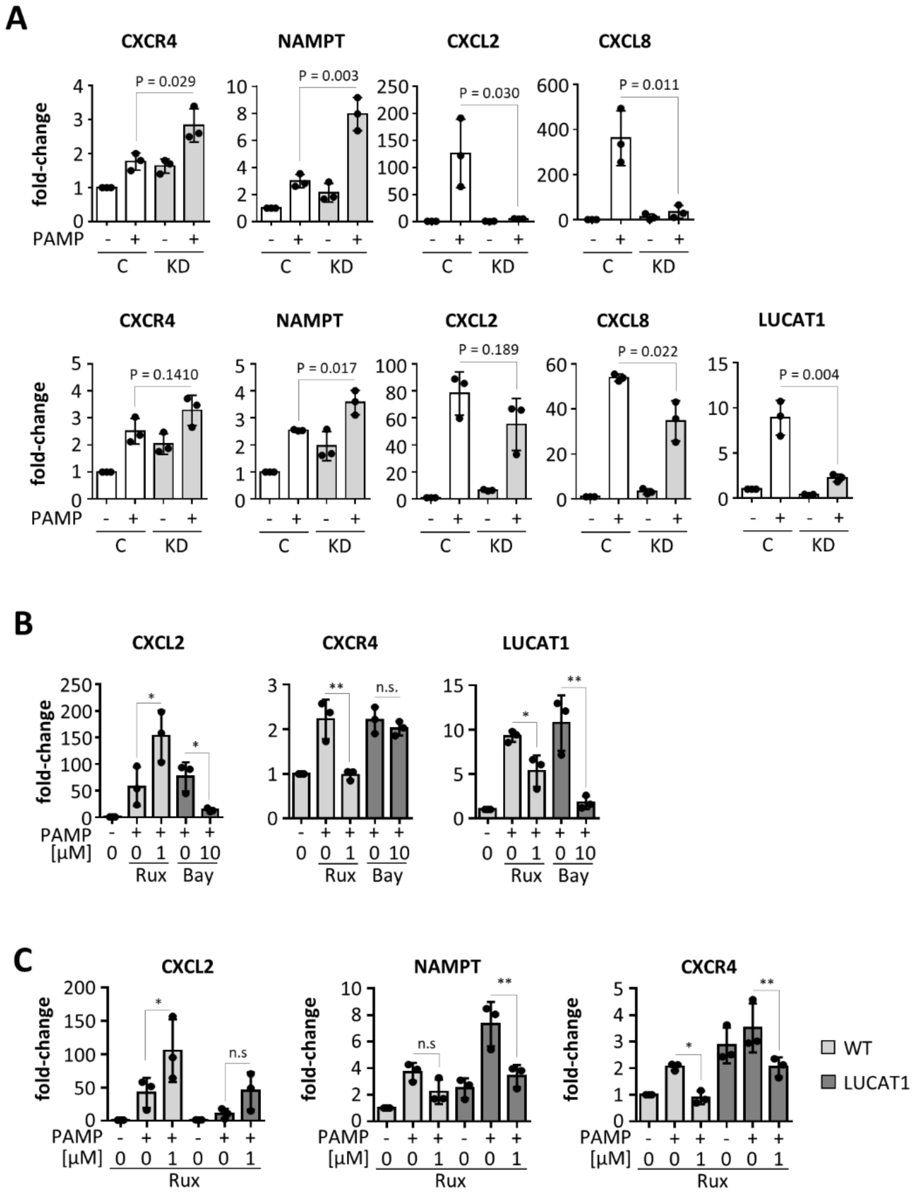

Fig. S7: Influence of LUCAT1 and pathway inhibitors on immune marker expression. A) qRT-PCR analysis of the expression of the indicated markers in control (C) and LUCAT1 knockdown (KD) THP1 monocytes, stimulated with LPS + polyI:C (PAMP) for 4 h or left untreated. Upper panel: results obtained with knockdown cell line from Fig. 3. Lower panel: results obtained with a second knockdown cell line, generated with an independent guideRNA (gRNA2, Table S7). Fold-changes relative to unstimulated control cells. B) Ruxolitinib and BAY-11-7082 sensitivity of CXCL2, CXCR4 and LUCAT1 (monocytes; PAMP = 4 h LPS + polyI:C; inhibitor pre-stimulation: 2 h). Fold-changes relative to unstimulated vehicle control. C) Rescue of CXCL2, NAMPT and CXCR4 dysregulation in LUCAT1 deficient THP1 cells (cell line from Fig. 3) upon 2 h Ruxolitinib treatment. A-C: One-way ANOVA, 3 independent experiments. * = P ≤ 0.05; ** P ≤ 0.01; n.s. = no significant difference.

A 5’/3’ RACE-PCR result

>PIRAT_cDNA_sequence

```
GAGGAACAGTCTTACTCTGTCACCCAGGCTGCAGTGTAGTGGTGTGATCACAGCTCACTGCAGCCTTGACCTCCTGGGC
TTAGGTGATCCTCCCACCCTAGCCTCCCATGTAGCTGGGACTAGAGGTATGTGCCACCTCACCTCCTTTTTTTCTTTTC
TTTTTCTTTTTTGGAGAGACAGATTCTTCTTATGTTGCTATTTTAAACTCCTGAACTCAAGTGATCCTCCTGCCTTGGC
CTCCCAAAGTGCTGGGATTACAGGTGTGAGACACTGCACCCTGCCCAAGCACCTCTGCTCCGTGCCATGCTCTTGGCTA
ATTGGAGTTGTGAAAGGCATGAGGATTCTGGTCATGGACTCAGCTCTCCCATAGGGTTCTGACACCAAAGCAATGGTCA
CAACAGTGAAAGGAAGAATCCATCTGGCCAGGCTCAGGTGGTTCAAGGCCTTCAGAATCTGCCTTGAGACTCTCACTGG
CTTTAGACTGAAAACCATCTTGGCCCCGTCCATCCGTGTAAGCAATTTAACGACAGCTTGCAAAGCACCGAGCTTTAAC
AGAAAGAAGAGATGAGCACAGCGCAAGAACTTGGACTCCAGAAGAGCTGCCTAACAGATTATTTTTCTGTGGCATTTCA
TGAGAACAAACGAAGTAGGAATTTTCCTTTTGTTTGTCTGGCCTTTGGCATCGTTTACTTTCTTTTTATTCTTCTGAAA
TGTACTTCGAGCCCTGGCAGCATTTCTGTCCTAAAATCTTATTGTCAGAGGTTTATTTTTCAGCTTTTCAAATCATATC
TGATAGAGTGAGTGTACTGCCTGGACTCATCACTTTACTTCAGAAGAAATACAGCTCACCCTTTAAATGACAATGGTGA
CTGTCCACATCTTTATGTTTTCTACACTGAAGTGGCAGGCTTCATTTAAAAATAATGTTTTCCCTCATCAAAAGAGAGC
TAGGGTAGAACCGTCAACTCTGCTGTTGTCTGGGTAGTGACCTAACACCCACGTTTTGGACAATCACTCACTGTCTTAT
ATTGGGTTTTCATTGCATGTAGGATAATTCTTTGTCAATGGTAGTTTTGTCAACCGTGATCTGAGGTAATGAGGTTTTC
TACTTTTGCTTGAAATTTTGAAAATATGCAAGCTTTAAACATTT
```

Fig. S8. Characterization of PIRAT cDNA sequence by RACE PCR. A) Full-length PIRAT sequence in human monocytes, reconstructed from Rapid Amplification of cDNA Ends (5’ and 3’) experiments and subsequent full-length PCR amplification and Sanger sequencing.

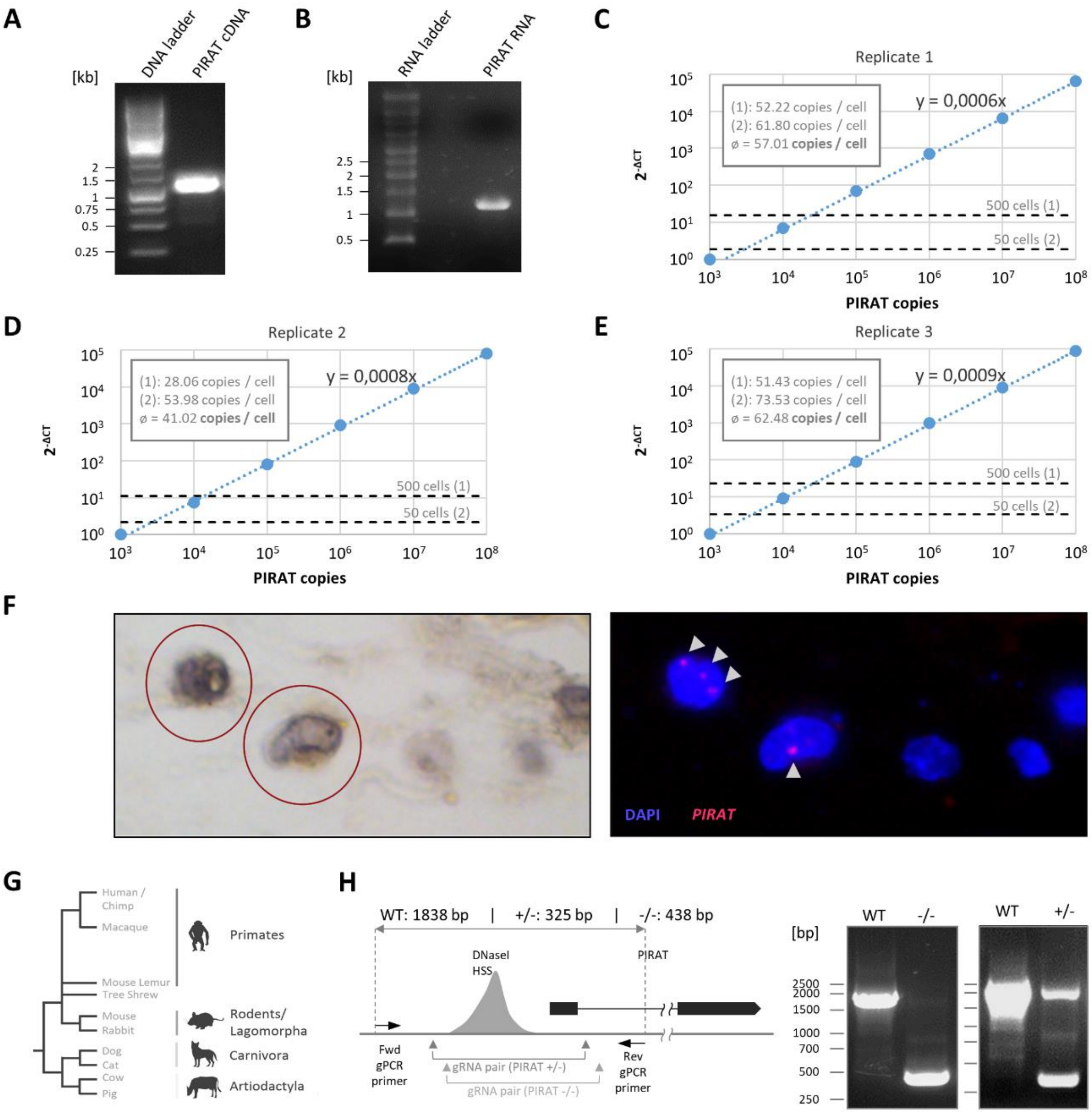

Fig. S9. Basic properties of lincRNA PIRAT. A) Full length PIRAT cDNA with 5' T7 promoter (PCR amplicon), separated on TBE agarose gel. Ladder: 1 kb Gene Ruler (Thermo Fisher). B) *In vitro* transcribed, column-purified PIRAT RNA (synthesized using DNA template shown in A), separated on MOPS/formaldehyde agarose gel. Ladder: Millennium RNA marker (Thermo Fisher). C-E) Same as Fig. 4C (PIRAT copy number enumeration by absolute, quantitative PCR), but showing the three independent experimental replicates separately, as well as linear regression formula. F) Left: hematoxylin staining of human lung slice. Circles indicate alveolar phagocytes. Right: RNA-FISH analysis of PIRAT subcellular localization in the same image. Nuclei counter-stained with DAPI. White arrows indicate PIRAT signal. G) Phylogenetic relationship of selected mammalian species and their respective orders. H) Left: schematic representation of guideRNA and genomic PCR primer binding sites in the PIRAT (LINC00211) locus. Expected Genomic PCR amplicon sizes for wild-type (WT), monoallelic (+/-) and biallelic knockouts (-/-) are indicated on the top. Right: Agarose gels showing genomic PCR amplicons from wild-type and PIRAT -/- and +/- THP1 cells.

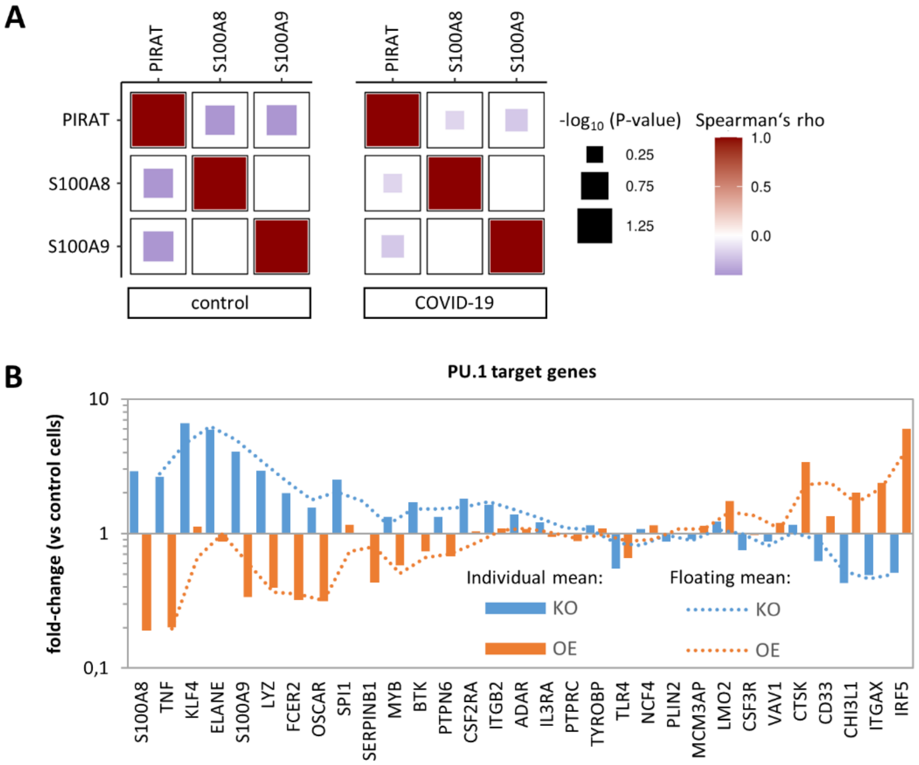

Fig. S10: Influence of PIRAT on PU.1 target gene expression. A) Single cell PIRAT, S100A8 and S100A9 co-expression analysis (across all cell types in control and COVID-19 patient scRNA-seq data shown in Fig. 2). Size of the filled squares indicates P-value and color indicates correlation coefficient (Spearman's rho), as indicated in the legends to the right. B) Fold-changes and floating mean of PU.1 target genes in PIRAT knockout (KO) and PIRAT overexpressing (OE), compared to wild-type THP1 cells (RNA-seq experiment from Fig. 4G; experimental replicate values averaged).

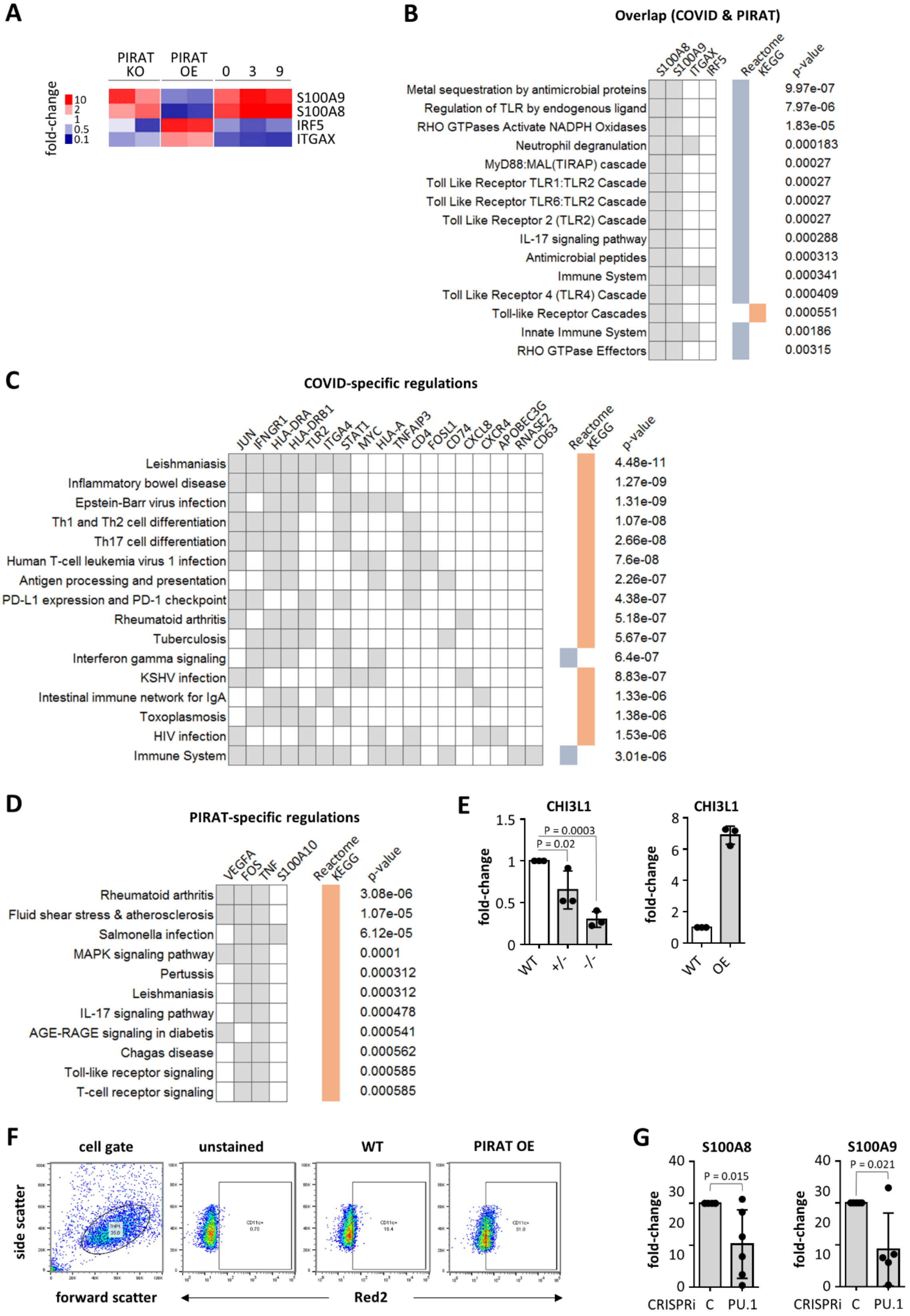
A
PIRAT KO
PIRAT OE
0
3
9
fold-change
10
2
1
0.5
0.1
S100A9
S100A8
IRF5
ITGAX
B
Overlap (COVID & PIRAT)
S100A8
S100A9
ITGAX
IRF5
Reactome
KEGG
p-value
Metal sequestration by antimicrobial proteins 9.97e-07
Regulation of TLR by endogenous ligand 7.97e-06
RHO GTPases Activate NADPH Oxidases 1.83e-05
Neutrophil degranulation 0.000183
MyD88:MAL(TIRAP) cascade 0.00027
Toll Like Receptor TLR1:TLR2 Cascade 0.00027
Toll Like Receptor TLR6:TLR2 Cascade 0.00027
Toll Like Receptor 2 (TLR2) Cascade 0.00027
IL-17 signaling pathway 0.000288
Antimicrobial peptides 0.000313
Immune System 0.000341
Toll Like Receptor 4 (TLR4) Cascade 0.000409
Toll-like Receptor Cascades 0.000551
Innate Immune System 0.00186
RHO GTPase Effectors 0.00315
C
COVID-specific regulations
JUN
IFNGR1
HLA-DRA
HLA-DRB1
TLR2
ITGA4
STAT1
MYC
HLA-A
TNFAIP3
CD4
FOSL1
CD74
CXCL8
CXCR4
APOBEC3G
RNASE2
CD63
Reactome
KEGG
p-value
Leishmaniasis 4.48e-11
Inflammatory bowel disease 1.27e-09
Epstein-Barr virus infection 1.31e-09
Th1 and Th2 cell differentiation 1.07e-08
Th17 cell differentiation 2.66e-08
Human T-cell leukemia virus 1 infection 7.6e-08
Antigen processing and presentation 2.26e-07
PD-L1 expression and PD-1 checkpoint 4.38e-07
Rheumatoid arthritis 5.18e-07
Tuberculosis 5.67e-07
Interferon gamma signaling 6.4e-07
KSHV infection 8.83e-07
Intestinal immune network for IgA 1.33e-06
Toxoplasmosis 1.38e-06
HIV infection 1.53e-06
Immune System 3.01e-06
D
PIRAT-specific regulations
VEGFA
FOS
TNF
S100A10
Reactome
KEGG
p-value
Rheumatoid arthritis 3.08e-06
Fluid shear stress & atherosclerosis 1.07e-05
Salmonella infection 6.12e-05
MAPK signaling pathway 0.0001
Pertussis 0.000312
Leishmaniasis 0.000312
IL-17 signaling pathway 0.000478
AGE-RAGE signaling in diabetis 0.000541
Chagas disease 0.000562
Toll-like receptor signaling 0.000585
T-cell receptor signaling 0.000585
E
CHI3L1
fold-change
P = 0.0003
P = 0.02
1.5
1
0.5
0
WT
+/-
-/-
CHI3L1
fold-change
8
6
4
2
0
WT
OE
F
cell gate
unstained
WT
PIRAT OE
side scatter
forward scatter
Red2
G
S100A8
fold-change
P = 0.015
40
30
20
10
0
CRISPRi
C
PU.1
S100A9
fold-change
P = 0.021
40
30
20
10
0
CRISPRi
C
PU.1

Fig. S11: Role of PIRAT in human monocytes. A) Heatmap showing fold-changes of genes regulated by PIRAT (RNA-seq; KO = knockout, OE = overexpression) and in monocyte populations 0, 3 and 9 during COVID-19 (scRNA-seq). Overlap of Venn diagram in Fig. 5A. B) Consensus Path DB KEGG and Reactome pathway analysis of genes regulated in COVID-19 and upon PIRAT expression-manipulation in THP1 cells (overlap of Venn diagram in Fig. 5A). Pathway terms, pertaining genes, pathway sources and p-values are shown. Grey fill color indicates that a given gene is included in the respective pathway. C) Same as B, but for genes regulated in COVID-19 only (Fig. 5A, left circle, without overlap). D) Same as B, but for genes regulated upon PIRAT expression manipulation only (Fig. 5A, right circle, without overlap). E) qRT-PCR analysis of CHI3L1 expression in wild-type and PIRAT-deficient (+/- and -/-), as well as in PIRAT over-expressing (OE) THP1 cells. Mean, individual replicate values and standard deviation based on three independent experiments are shown. One-way ANOVA. F) Representative dot plots illustrating the gating strategy used for FACS-quantification of CD11c (ITGAX) positive THP1 cells (Fig. 5F). G) qRT-PCR analysis of expression changes of S100A8 and S100A9 upon PU.1- compared to control-CRISPRi (THP1 cells. ≥ 5 experimental replicates. Replicate values, mean and standard deviation are shown. Two-tailed Student's t-test.).

A

TFs driving genes up-regulated after LUCAT1 knockdown

Index	Name	P-value	Adjusted p-value	Odds Ratio	Combined score
1	STAT3 CHEA	0.00003694	0.001773	68.34	697.48
2	IRF1 ENCODE	0.004578	0.1099	25.20	135.74
3	IRF8 CHEA	0.04740	0.2018	23.66	72.14
4	ZC3H11A ENCODE	0.05046	0.2018	22.17	66.21
5	ERG CHEA	0.05618	0.2074	19.83	57.09
6	STAT3 ENCODE	0.03178	0.2018	8.88	30.64
7	ZNF384 ENCODE	0.03218	0.2018	8.82	30.31
8	STAT5A ENCODE	0.08950	0.2685	12.17	29.37
9	GATA1 CHEA	0.03873	0.2018	7.94	25.83
10	UBTF ENCODE	0.02219	0.2018	6.77	25.77

TFs driving genes down-regulated after LUCAT1 knockdown

Index	Name	P-value	Adjusted p-value	Odds Ratio	Combined score
1	RELA ENCODE	0.002450	0.1397	8.12	48.83
2	FOSL2 ENCODE	0.02289	0.5989	9.27	35.01
3	STAT5A ENCODE	0.03152	0.5989	7.77	26.86
4	KAT2A ENCODE	0.1026	0.7175	9.72	22.12
5	PPARD CHEA	0.04545	0.6157	6.33	19.55
6	IRF8 CHEA	0.1356	0.7175	7.19	14.37
7	BHLHE40 ENCODE	0.06481	0.6157	5.16	14.11
8	ZC3H11A ENCODE	0.1439	0.7175	6.74	13.07
9	CEBPD ENCODE	0.05622	0.6157	3.76	10.83
10	ESR1 CHEA	0.1694	0.7175	5.63	10.00

TFs driving genes up- or down-regulated after PIRAT knockout

Index	Name	P-value	Adjusted p-value	Odds Ratio	Combined score
1	IRF8 CHEA	0.02398	0.09593	55.21	205.96
2	SPI1 CHEA	0.1951	0.2966	5.98	9.78
3	TP63 CHEA	0.2518	0.2966	4.43	6.12
4	SUZ12 CHEA	0.2966	0.2966	3.63	4.41

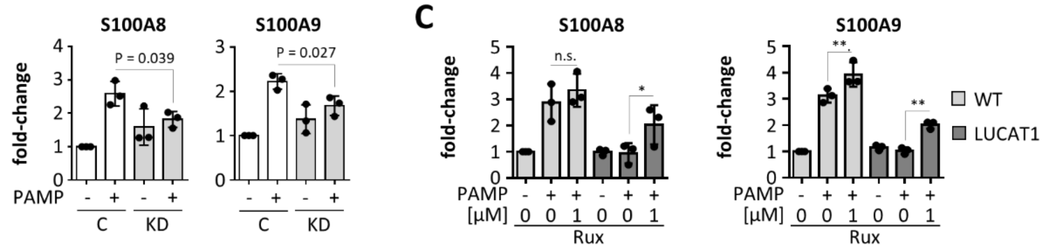

Fig. S12: Impact of LUCAT1 and PIRAT on genes regulated in monocytes during COVID-19. A) Prediction of transcription factors (TFs) driving the indicated sets of genes from Fig. 5H. Predictions were done using the ENCODE and ChEA Consensus TFs tab at Enrichr (https://maayanlab.cloud/Enrichr/). Top 10 transcription factors are shown (for the PIRAT controlled gene set only 4 transcription factors were identified). Transcription factor predictions supported by experimental data in the present study are highlighted. B) qRT-PCR analysis of the expression of S100A8 and S100A9 in control (C) and LUCAT1 knockdown (KD) THP1 monocytes, stimulated with LPS + polyI:C (PAMP) for 4 h or left untreated. Results obtained with second knockdown cell line, generated with guideRNA 2 (gRNA2, Table S7). Fold-changes relative to unstimulated control cells. C) Restoration of S100A8/9 expression in LUCAT1 KD cells upon 2 h Ruxolitinib and 4 h LPS + polyI:C treatment. B-C: Three independent replicates, One-way ANOVA. * = P ≤ 0.05; ** = P ≤ 0.01.

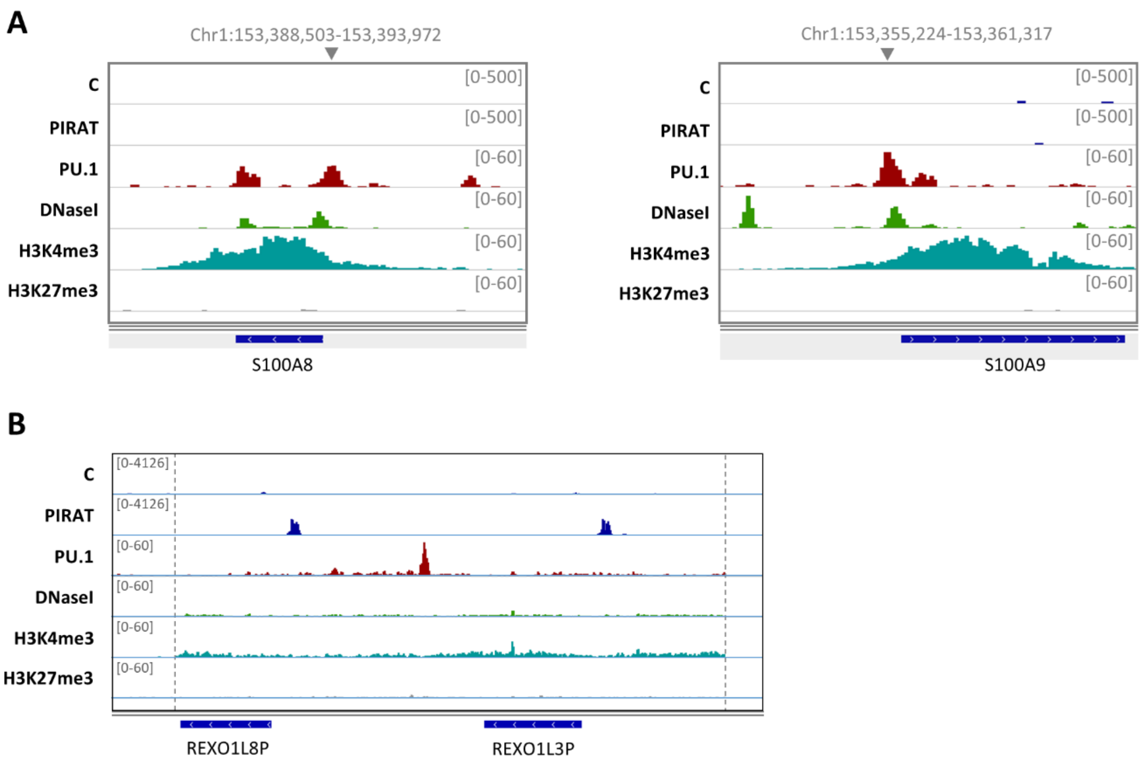

Fig. S13. Characterization of PIRAT interplay with PU.1 at the DNA level. A) IGV plots showing PIRAT ChIRP-seq, PU.1 and histone H3 ChIP-seq and DNaseI-seq coverage in the S100A8 and A9 loci. Grey triangles indicate the assumed PU.1 proximal promoter binding sites. Track height is indicated in brackets. **B)** IGV plot showing control (C) and PIRAT ChIRP-seq and matched CD14+ monocyte PU.1 ChIP-, DNaseI-, and histone-3 ChIP-seq coverage in the REXO1L pseudogene locus (**Fig. 6F** zoomed image, spanning REXO1L8P and REXO1L3P). Track-height indicated in brackets.

A

B

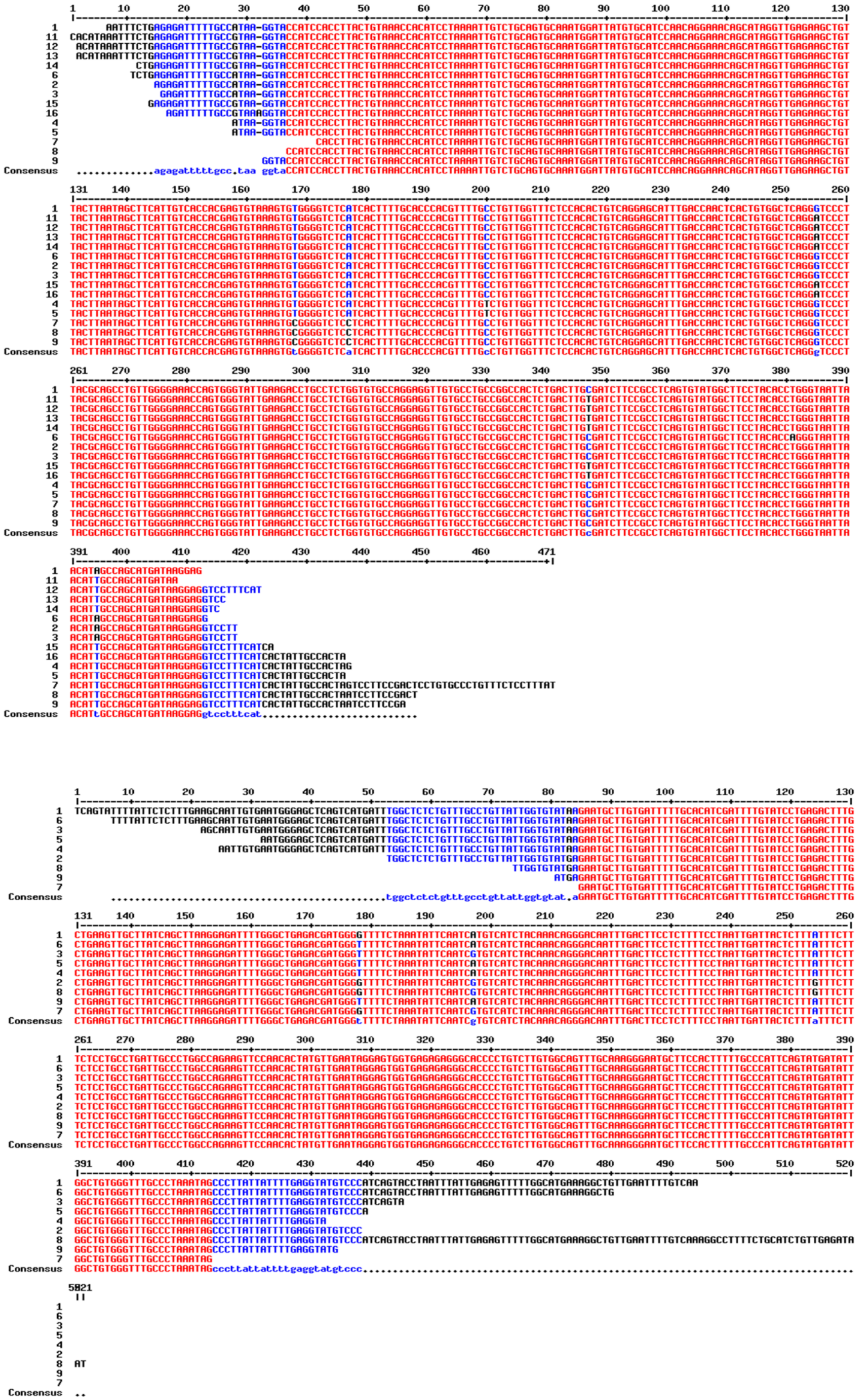

Fig. S14: Alignment of REXO1LP sequences. A) Multiple sequence alignment (http://multalin.toulouse.inra.fr/multalin/) of PIRAT binding sites in the REXO1LP locus (peaks from Fig. 6F). B) Same as A, but for PU.1 peaks from Fig. 6F / S13B.

A

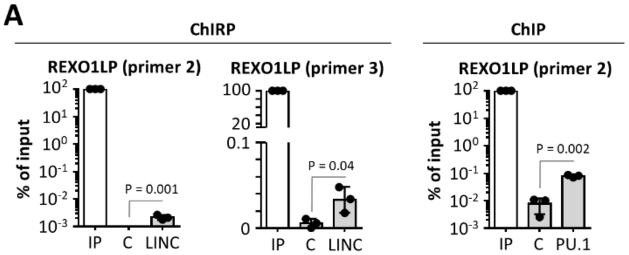

B

REXO1LP (primer 3)

Variant	%	# (D1)	# (D2)	# (D3)
A/A/A/T/C	18.5	2	2	1
G/G/C/A/T	44.4	6	4	2
G/G/C/T/T	18.5	1	3	1
G/G/C/A/C	18.5			5

C

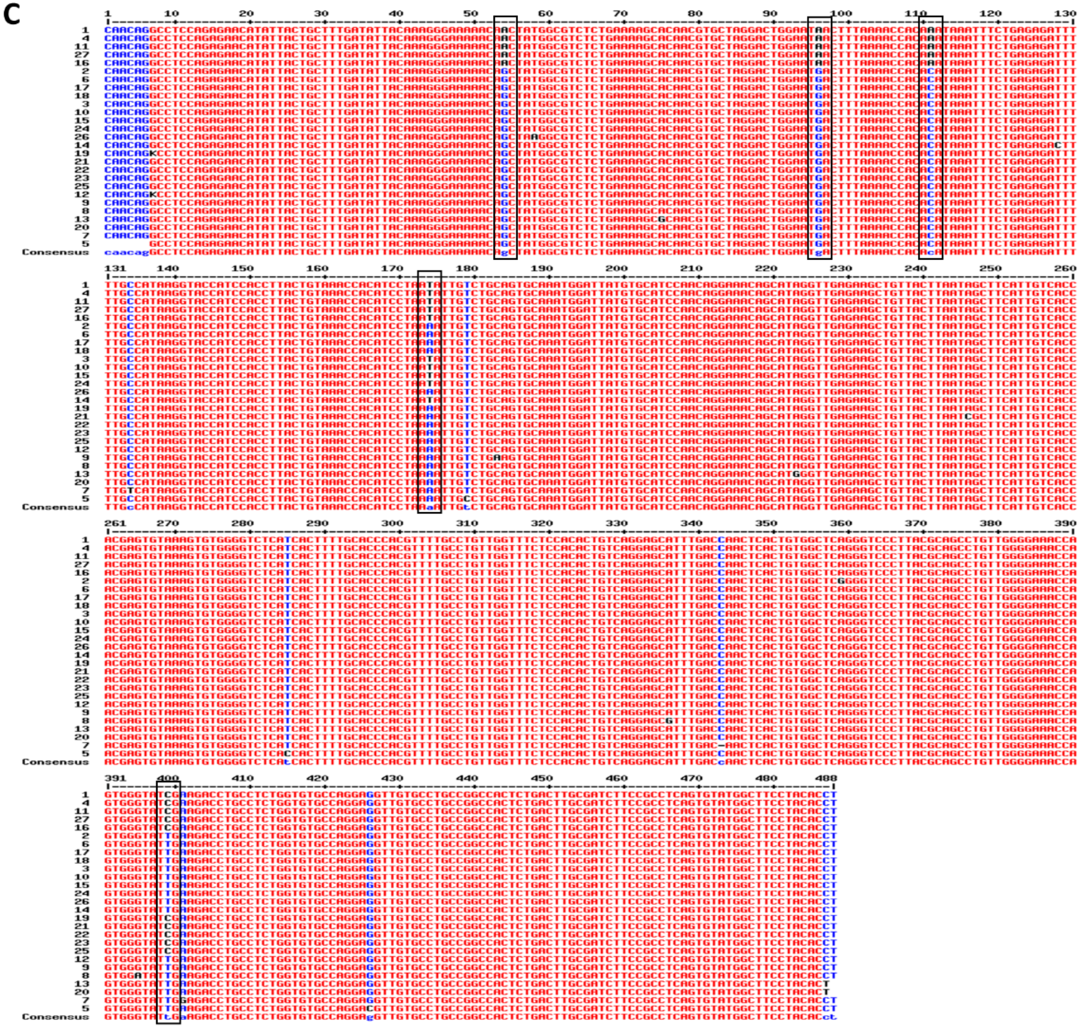

D

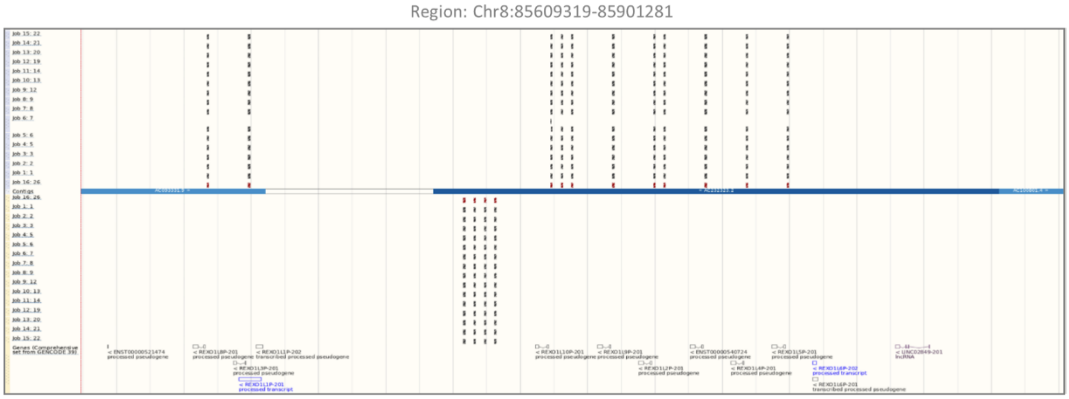

Fig. S15: Characterization of PIRAT binding sites in the REXO1LP locus. A) Same as Fig. 6G and H, but with additional primer pairs. B) Enumeration of single nucleotide variants discriminating cloned and Sanger-sequenced PCR amplicons of the PIRAT binding sites amplified using the same primers as in panel A (elution fraction, “LINC”), but with Advantage 2 proof-reading polymerase. % indicates percentage of variant among all Sanger sequences. # indicates the total number a given variant was detected in experiments performed with ChIRP DNA from monocytes isolated from blood samples of three different donors (D1, D2, D3). C) Aligned Sanger sequences from analysis performed in B, with enumerated nucleotide variants highlighted. D) ENSEMBL Genome Browser view of all obtained BLAST hits for Sanger sequences from C (human GRCh38 genome).

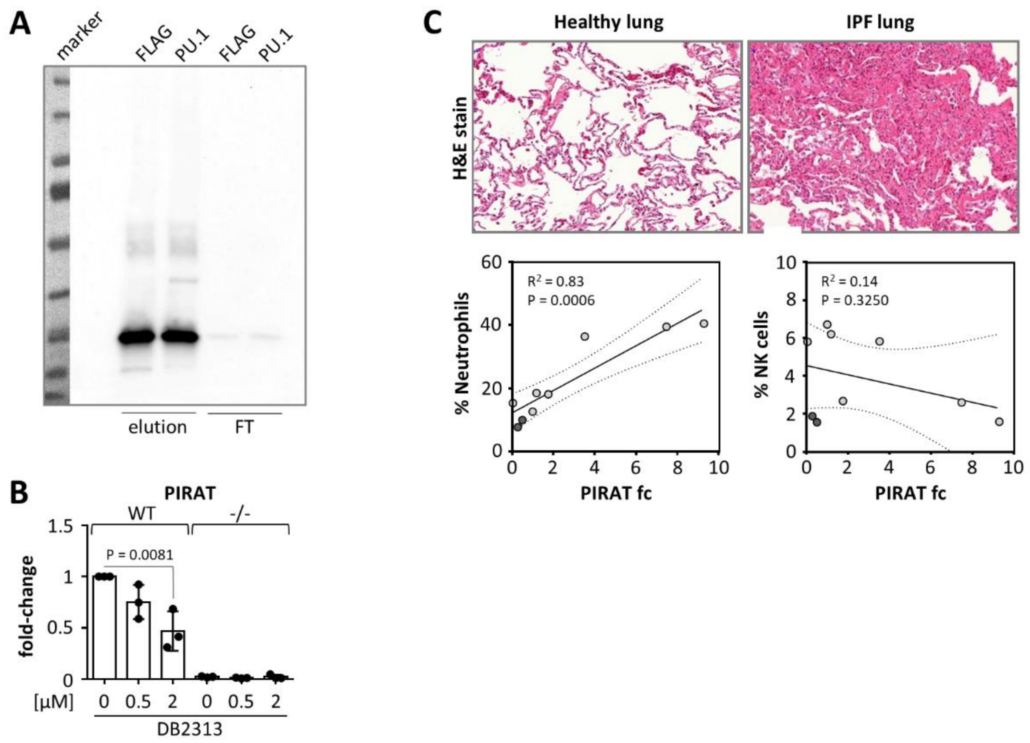

Fig. S16: PU.1 dependency of PIRAT and relevance to IPF. A) Full-scan of PU.1 CoIP Western blot shown in Fig. 7A (FT = flow-through fractions). B) qRT-PCR analysis of PIRAT expression after treatment of wild-type (WT) and PIRAT knockout (-/-) THP1 monocytes with the PU.1 inhibitor DB2313 (concentrations indicated) for 4 h. Three independent experiments and One-way ANOVA. C) Top: Representative H&E-stained sections of human healthy and late-stage IPF lung tissue. Bottom: Pearson correlation of neutrophil or NK cell percentage with PIRAT fold-change (compared to IPF lung # 3).

Table S1. Myeloid and lymphoid cell specific lincRNAs identified in the current study (RPKM and standard-deviation are shown).

Alias	Monocytes	Granulocytes	B cells	NK cells	CD4 T cells	CD8 T cells
AC064805.1	4.58 ± 1.19	0.23 ± 0.20	0.02 ± 0.03	0.09 ± 0.13	0.01 ± 0.01	0.00 ± 0.00
AP001257.1	4.03 ± 1.73	0.01 ± 0.02	0.02 ± 0.04	0.02 ± 0.03	0.01 ± 0.02	0.03 ± 0.03
AP003774.3	3.92 ± 1.83	0.06 ± 0.10	0.02 ± 0.04	0.04 ± 0.07	0.01 ± 0.01	0.01 ± 0.01
LINC02285	3.69 ± 0.65	2.11 ± 1.06	1.20 ± 0.44	1.62 ± 0.56	0.92 ± 0.18	1.25 ± 0.19
AC097504.2	3.53 ± 0.45	0.00 ± 0.00	0.00 ± 0.00	0.00 ± 0.00	0.00 ± 0.00	0.00 ± 0.00
LUCAT1	2.49 ± 1.62	23.97 ± 15.61	0.02 ± 0.01	0.03 ± 0.02	0.02 ± 0.02	0.03 ± 0.03
LINC01506	0.57 ± 0.09	9.27 ± 2.93	0.03 ± 0.01	0.00 ± 0.00	0.00 ± 0.00	0.00 ± 0.00
AC007342.5	0.04 ± 0.05	8.38 ± 1.80	0.01 ± 0.02	0.00 ± 0.00	1.18 ± 0.67	1.27 ± 0.66
PIRAT	0.71 ± 0.47	4.63 ± 1.46	0.00 ± 0.00	0.04 ± 0.04	0.02 ± 0.03	0.02 ± 0.03
LINC00921	0.52 ± 0.24	3.96 ± 1.01	0.14 ± 0.15	1.24 ± 1.30	0.33 ± 0.26	0.30 ± 0.29
AC104971.4	0.06 ± 0.11	0.00 ± 0.00	7.52 ± 0.22	0.04 ± 0.04	0.50 ± 0.20	0.23 ± 0.20
AC009686.2	0.00 ± 0.00	0.00 ± 0.00	5.18 ± 1.64	0.16 ± 0.28	0.16 ± 0.15	0.37 ± 0.30
AC006033.2	1.17 ± 0.11	0.10 ± 0.01	0.02 ± 0.04	9.40 ± 1.20	0.02 ± 0.02	0.63 ± 0.22
AC093323.2	0.16 ± 0.25	0.17 ± 0.10	0.06 ± 0.04	6.48 ± 1.97	1.35 ± 0.81	2.60 ± 1.17
LINC00861	0.21 ± 0.34	0.08 ± 0.13	0.05 ± 0.07	10.34 ± 5.29	10.49 ± 3.64	9.21 ± 2.00
LINC01550	0.03 ± 0.05	0.01 ± 0.02	0.00 ± 0.00	0.02 ± 0.01	2.20 ± 0.52	1.97 ± 0.21
LINC02295	0.00 ± 0.00	0.00 ± 0.00	0.00 ± 0.00	0.00 ± 0.00	0.81 ± 0.90	0.46 ± 0.28
LINC02361	0.72 ± 0.25	0.48 ± 0.28	0.61 ± 0.43	2.30 ± 0.29	9.96 ± 3.44	5.83 ± 1.50
LINC02273	0.03 ± 0.05	0.01 ± 0.02	0.06 ± 0.02	0.27 ± 0.05	5.50 ± 0.67	3.01 ± 1.35
LINC02446	0.15 ± 0.26	0.02 ± 0.02	0.01 ± 0.01	0.40 ± 0.36	0.04 ± 0.05	15.72 ± 7.31

Table S2. COVID patient characteristics (cohort 1). Red: patient PBMCs analyzed by single cell RNA-seq.

Patient Nr.	Gender	Age	Patient group (WHO grade)
1	Female	68	Control
2	Female	46	Control
3	Male	92	Control
4	Female	47	Control
5	Male	74	Control
6	Male	65	Control
7	Male	68	Control
8	Male	83	COVID-19 (> 4)
9	Male	88	COVID-19 (> 4)
10	Male	78	COVID-19 (3)
11	Female	72	COVID-19 (8)
12	Male	70	COVID-19 (8)
13	Male	49	COVID-19 (8)
14	Female	52	COVID-19 (5)
15	Male	55	COVID-19 (4)
16	Male	59	COVID-19 (4)
17	Female	42	COVID-19 (2)
18	Female	71	COVID-19 (4)
19	Male	55	COVID-19 (4)

Table S3. COVID patient characteristics (cohort 2). ICU = intensive care unit.

Patient Nr.	Gender	Age	Patient group (intensive care unit)
1	Female	86	Control
2	Female	65	Control
3	Male	44	Control
4	Male	42	Control
5	Female	80	Control
6	Female	72	Control
7	Female	26	Control
8	Male	41	Control
9	Male	28	Control
10	Male	70	Control
11	Male	28	Control
12	Male	82	COVID-19 (ICU)
13	Female	58	COVID-19
14	Male	52	COVID-19
15	Male	44	COVID-19 (ICU)
16	Male	66	COVID-19 (ICU)
17	Male	23	COVID-19 (ICU)
18	Male	82	COVID-19 (ICU)

Table S4. List of direct PU.1 target genes.

Gene ID	Name
ENSG00000140678	ITGAX
ENSG00000160255	ITGB2
ENSG00000011600	TYROBP
ENSG00000066336	SPI1
ENSG00000147872	PLIN2
ENSG00000111679	PTPN6
ENSG00000104921	FCER2
ENSG00000232810	TNF
ENSG00000128604	IRF5
ENSG00000170909	OSCAR
ENSG00000160294	MCM3AP
ENSG00000185291	IL3RA
ENSG00000143387	CTSK
ENSG00000136826	KLF4
ENSG00000135363	LMO2
ENSG00000136869	TLR4
ENSG00000105383	CD33
ENSG00000021355	SERPINB1
ENSG00000105810	CDK6
ENSG00000150337	FCGR1A
ENSG00000119535	CSF3R
ENSG00000198223	CSF2RA
ENSG00000163220	S100A9
ENSG00000143546	S100A8
ENSG00000118513	MYB
ENSG00000090382	LYZ

Table S5. Significant PIRAT ChIRP-seq peaks. Chr = chromosome. GRCh38 genome.

Chr	Region	Peak end	Peak center	Peak length	Peak shape score	P-value	Nearest gene
8	85723057-85723461	85723461	85723258	405	9.742	9.98E-23	REXO1L2P
8	85807151-85807553	85807553	85807353	403	9.571	5.30E-22	REXO1L4P
8	85794959-85795360	85795360	85795160	402	9.484	1.22E-21	AC232323.1
8	85647089-85647497	85647497	85647297	409	8.983	1.31E-19	REXO1L8P
8	85659279-85659686	85659686	85659486	408	8.966	1.54E-19	REXO1L1P
8	85819338-85819739	85819739	85819539	402	8.959	1.64E-19	REXO1L5P
8	85755321-85755750	85755750	85755550	430	7.471	3.98E-14	REXO1L10P
8	85767511-85767922	85767922	85767722	412	7.150	4.34E-13	REXO1L9P
8	85782785-85783198	85783198	85782998	414	7.108	5.89E-13	REXO1L2P
13	113079768-113080178	113080178	113079969	411	6.610	1.92E-11	MCF2L
8	85729231-85729637	85729637	85729437	407	6.185	3.10E-10	REXO1L11P
8	85726132-85726551	85726551	85726351	420	6.062	6.71E-10	REXO1L11P
8	85732308-85732721	85732721	85732521	414	6.020	8.70E-10	REXO1L11P
8	85752207-85752609	85752609	85752408	403	5.965	1.22E-09	REXO1L10P
8	85779682-85780091	85780091	85779883	410	5.909	1.71E-09	REXO1L2P
8	85749126-85749545	85749545	85749327	420	5.827	2.81E-09	REXO1L10P
14	105702998-105703418	105703418	105703218	421	5.260	7.19E-08	ELK2AP
16	33338417-33338820	33338820	33338620	404	4.856	5.98E-07	AC141257.2
16	33339241-33339719	33339719	33339519	479	4.415	5.04E-06	AC141257.3
16	33336089-33336575	33336575	33336290	487	4.297	8.63E-06	AC141257.2
16	33491258-33491859	33491859	33491459	602	3.659	0.00012	AC136944.5
16	33337166-33337581	33337581	33337367	416	3.598	0.00016	AC141257.3
16	33337588-33338034	33338034	33337789	447	3.590	0.00016	AC141257.3
1	125182192-125182673	125182673	125182393	482	3.589	0.00016	No annotations (Chr 1q12)
2	37827524-37827925	37827925	37827725	402	3.490	0.00024	PIRAT
1	143193471-143193932	143193932	143193732	462	3.452	0.00027	TMEM128
1	143262376-143262850	143262850	143262650	475	3.419	0.00031	TMEM128
11	69872120-69872630	69872630	69872430	511	3.340	0.00041	FGF3
1	125179028-125179431	125179431	125179229	404	3.184	0.00072	No annotations (Chr 1q12)
12	124914336-124914767	124914767	124914567	432	3.042	0.00117	No annotations (Chr 1q12)
3	93470371-93470773	93470773	93470573	403	3.026	0.00123	REXO1L2P
8	85723057-85723461	85723461	85723258	405	9.742	9.98E-23	REXO1L4P

Table S6. Characteristics of control and pulmonary infection patients. n.d. = causative pathogen not determined.

Patient Nr.	Gender	Age	Patient group (type of infection)
1	male	53	control
2	male	49	control
3	male	38	control
4	female	71	control
5	female	49	control
6	male	62	control
7	male	28	control
8	male	29	control
9	female	27	control
10	male	64	control
11	female	25	infection (bacterial)
12	male	45	infection (bacterial)
13	female	64	infection (bacterial)
14	female	19	infection (fungal)
15	female	64	infection (fungal)
16	female	42	infection (fungal)
17	female	68	infection (n.d.)
18	female	59	infection (n.d.)
19	female	52	infection (n.d.)
20	female	58	infection (n.d.)
21	female	73	infection (n.d.)
22	female	61	infection (polymicrobial)

Table S7. PCR and sequencing oligonucleotides used in the present study.

Target (purpose)	Oligo name	Oligo sequence
SparQ MCS (Sanger sequencing)	OBS-0659	Fwd: AGGAGGATTTGATATTCACCTG
	OBS-0660	Rev: ACCTTCTCTAGGCACCCG
pX458 (Sanger sequencing)	OBS-0842	Fwd: CTGGCCTTTTGCTCACATGT
	OBS-0843	Rev: GTCTGCAGAATTGGCGCAC
hPIRAT (qRT-PCR)	OBS-0808	Fwd: TGAGTGTACTGCCTGGACTCATC
	OBS-0809	Rev: TAAATGAAGCCTGCCACTTCAG
hLUCAT1 (qRT-PCR)	OBS-0849	Fwd: ACCATGTGTCAAGCTCGGATTG
	OBS-0850	Rev: TTGTGGTCTCTGGTGCCAAG
AC064805.1 (qRT-PCR)	OBS-0899	Fwd: TAATAAGCAGCAATTGCAGTTCC
	OBS-0900	Rev: TATCTGCTCCTGAGGCAGAGG
LINC02295 (qRT-PCR)	OBS-0935	Fwd: AAGCTGTGGCTGTTGTCAGC
	OBS-0936	Rev: ACACTTGTCTCAGTAGGCCTGG
LINC02446 (qRT-PCR)	OBS-0937	Fwd: TGTCACCTGTGGACAACTTGC
	OBS-0938	Rev: TTATCTTGACCAGGTGCGAGAC
LINC00861 (qRT-PCR)	OBS-0907	Fwd: AACACTGAGCAATCCTGACCTG
	OBS-0908	Rev: TATCGGTCCTCCACTCTTGTTC
hS100A8 (qRT-PCR)	OBS-2077	Fwd: AGACTGTAGCAACTCTGGCAG
	OBS-2078	Rev: TCCAGCTCGGTCAACATGATG
hS100A9 (qRT-PCR)	OBS-2198	Fwd: ACCAATACTCTGTGAAGCTGG
	OBS-2199	Rev: TCCTCGAAGCTCAGCTGCTTG
hIRF5 (qRT-PCR)	OBS-3742	Fwd: ATGCCACAAGGCATGGTCC
	OBS-3743	Rev: TCGTAGATGAGGCGGAAGTC
hCHI3L1 (qRT-PCR)	OBS-2179	Fwd: AGGGACCCTTGCCTACTATGA
	OBS-2180	Rev: TGGAAGTCATCCAGGTCCAGG
hITGAX (qRT-PCR)	OBS-2275	Fwd: AAGCTGACAGACGTGGTCATC
	OBS-2276	Rev: ATACTGCAGCCTGGAGGAGAG
hCXCL2 (qRT-PCR)	OBS-534	Fwd: CCTGCAGGGAATTCACCTCA
	OBS-535	Rev: CCTTCCTTCTGGTCAGTTGG
hCXCR4 (qRT-PCR)	OBS-3512	Fwd: AGAACCAGCGGTTACCATGG
	OBS-3513	Rev: TGACCAGGATGACCAATCCATTG
hNAMPT (qRT-PCR)	OBS-1458	Fwd: GGTTACAAGTTGCTGCCACC
	OBS-1459	Rev: AGCAAACCTCCACCAGAACC

hCXCL8 (qRT-PCR)	OBS-0017	Fwd: ACTGAGAGTGATTGAGAGTGGAC
	OBS-0018	Rev: AACCCTCTGCACCCAGTTTTC
hIL6 (qRT-PCR)	OBS-15	Fwd: AATTCGGTACATCCTCGACGG
	OBS-16	Rev: TTGGAAGGTTCAGGTTGTTTTCT
hPU.1 (qRT-PCR)	OBS-2399	Fwd: AGAGCCATAGCGACCATTACTG
	OBS-2400	Rev: ATCTGCTCCAGCTCCATGTG
hGAPDH (qRT-PCR)	OBS-0430	Fwd: CCACATCGCTCAGACACCAT
	OBS-0431	Rev: CGCAACAATATCCACTTTACCAGAG
hMALAT1 (qRT-PCR)	OBS-814	Fwd: AGGTGCTACACAGAAGTGGATTCAG
	OBS-815	Rev: CTTCCCGTACTTCTGTCTTCCAGT
hU6 (qRT-PCR)	OBS-0712	Fwd: GCTTCGGCAGCACATATACTAAAAT
	OBS-0713	Rev: ATATGGAACGCTTCACGAATTTG
hS100A8 promoter (qRT-PCR)	OBS-3211	Fwd: AGCATCCACTTCCTATTCCTGC
	OBS-3212	Rev: TAAGGATTTGGGTAGCATGGAGG
hS100A9 promoter (qRT-PCR)	OBS-3213	Fwd: TGAACTAAACAACCAGCTTCCTCC
	OBS-3214	Rev: TGAGCAGTGTGGTAATGCTGC
hREXOL1P PIRAT peak primer 1 (qRT-PCR)	OBS-3215	Fwd: ACAGTGAGTTGGTCAAATGCTCC
	OBS-3216	Rev: ACAGCATAGGTTGAGAAGCTGTTAC
hREXOL1P PIRAT peak primer 2 (qRT-PCR)	OBS-3221	Fwd: TTCTCCACACTGTCAGGAGC
	OBS-3222	Rev: AGGTGTAGGAAGCCATACACTG
hREXOL1P PIRAT peak primer 3 (qRT-PCR)	OBS-3734	Fwd: CAACAGGCCTCCAGAGAAC
	OBS-3222	Rev: AGGTGTAGGAAGCCATACACTG
hREXOL1P PU.1 peak primer 1 (qRT-PCR)	OBS-3202	Fwd: CCACAGCCAATATCATACTGAATGG
	OBS-3203	Rev: ACAAACAGGGACAATTTGACTTCCTC
hREXOL1P PU.1 peak primer 2 (qRT-PCR)	OBS-3204	Fwd: GCTGAAGTTGCTTATCAGCTTAAGG
	OBS-3205	Rev: ACATAGTGTTGGAACTTCTGGCC
hPIRAT (genomic PCR)	OBS-1895	Fwd: GCATCTGCATGGCAGAGTTC
	OBS-1239	Rev: TATGGCTCTTGCAATTAATCCTG
hPIRAT 3’ (RACE)	OBS-1058	AGAGTGAGTGTACTGCCTGGACTCATCAC
hPIRAT 5’ (RACE)	OBS-1059	ACATAAAGATGTGGACAGTCACCATTGTC
hPIRAT (FL cloning)	OBS-1356	Fwd: atcggaTTCGAAGAGGAACAGTCTTACTCTGTCAC
	OBS-1357	Rev: tccgatGCGGCCGCAAATGTTTAAAGCTTGCATATTTTC
hPIRAT (in vitro transcription PCR)	OBS-1898	Fwd: GTTTTTTTTAATACGACTCACTATAGGAGGAACAGTCTTACTCTGTCAC
	OBS-1899	Rev: AAATGTTTAAAGCTTGCATATTTTC
hPIRAT (gRNA -/-)	LS-1	Sense strand: GATGAGTCTAACGTGCACCC

	LS-2	Antisense strand: GAGAGTTATAACATAATGGT
hPIRAT (gRNA +/-)	LS-3	Sense strand: ACGGATGGCCTTGGTCACCC
	LS-4	Antisense strand: TTACATGAATAGACAGCTAG
hLUCAT1 (gRNA 1)	OBS-2092	Sense strand: CACCGAAGCTCGGATTGCCTTAGAC
	OBS-2093	Antisense strand: AAACGTCTAAGGCAATCCGAGCTTC
hLUCAT1 (gRNA 2, partial knockdown)	OBS-2094	Sense strand: CACCGCGAGCTTGACACATGGTTTC
	OBS-2095	Antisense strand: AAACGAAACCATGTGTCAAGCTCGC
hPU.1 (gRNA)	OBS-2281	Sense strand: CACCGAAATCTCTTGCGCTACATAC
	OBS-2282	Antisense strand: AAACGTATGTAGCGCAAGAGATTTC

Table S8. Antibodies used in the present study.

Specificity	Source	Class	Conjugate	Supplier	Catalog nr.	Application
PU.1 (A7)	mouse	IgG1	-	Santa Cruz	sc-365208	Western Blot, IP
PU.1 (C3)	mouse	IgG	-	Santa Cruz	sc-390405	IP
Flag	mouse	IgM2	-	Sigma-Aldrich	F1804	IP
Anti-mouse	goat	IgG	HRP	Santa Cruz	sc-2005	Western Blot
CD14	mouse	IgG1	FITC	eBioscience	11-0149-42	FACS
CD66b	mouse	IgM	APC	eBioscience	17-0666-42	FACS
CD4	mouse	IgG2b	PE	eBioscience	12-0048-41	FACS
CD8	mouse	IgG1	FITC	eBioscience	11-0087-42	FACS
CD19	mouse	IgG1	FITC	eBioscience	11-0199-41	FACS
CD56	mouse	IgG	APC	eBioscience	17-0567-41	FACS
CD11c	mouse	IgG1	APC	eBioscience	17-0116-42	FACS

Table S9. ChIRP oligonucleotides used in the present study.

Target (purpose)	Oligo name	Oligo sequence
PIRAT	OBS-2890	TCCAATTAGCCAAGAGCATG
PIRAT	OBS-2891	CTTTCACTGTTGTGACCATT
PIRAT	OBS-2892	GTTTTCAGTCTAAAGCCAGT
PIRAT	OBS-2893	CTCATCTCTTCTTTCTGTTA
PIRAT	OBS-2894	ATGCCAAAGGCCAGACAAAC
PIRAT	OBS-2895	AAAGTGATGAGTCCAGGCAG
PIRAT	OBS-2896	CTGCCACTTCAGTGTAGAAA
PIRAT	OBS-2897	TGTCCAAAACGTGGGTGTTA
PIRAT	OBS-2898	ATTACCTCAGATCACGGTTG
None (control ChIRP)	OBS-2899	CACTATGGAAAGGCGGCTTC
None (control ChIRP)	OBS-2900	GATTTCGGTCTGTACGGCTA
None (control ChIRP)	OBS-2901	TTACATGGTCCTAATCGGCT
None (control ChIRP)	OBS-2902	GCTGTTACCTTCCACGCCGG
None (control ChIRP)	OBS-2903	ATACGATCGGACAGCCTTGT
None (control ChIRP)	OBS-2904	TGCACAATTGATGTTCCGAT
None (control ChIRP)	OBS-2905	GACGCCTAGACGTATACTAG
None (control ChIRP)	OBS-2906	GTGTGTGCTATTAGAAGCGG
None (control ChIRP)	OBS-2907	AAGCGACCCTGACAGTGCGA
None (control ChIRP)	OBS-2908	AGCAAACACGTCGAGCAAAT

Supplementary Methods

Cell culture and human biomaterial

Human peripheral blood mononuclear cells from healthy donors (control patient cells see below) were isolated from buffy coats (transfusion medicine department, UKGM Giessen). Buffy coats were de-identified prior to further use. Leukocyte populations were purified from buffy coats using Lymphoprep gradient medium (Stemcell Technologies) and MACS-purification (Miltenyi CD14-, CD4-, CD8-, CD45RO-, CD19-, CD56- and CD66b-beads). CD4 and CD8 T-cells were separated into CD45RO-positive and -negative populations, respectively. Blood-derived macrophages and dendritic cells were obtained by cultivating monocytes in the presence of 100 ng / ml GM-CSF or 50 ng / ml GM-CSF, 20 ng / ml IL-4 (Preprotech), respectively, in X-Vivo 15 medium (Lonza), containing 5 % fetal calf serum (FCS, Biochrom) for 7 days. Cell populations shown in Fig. 2H were isolated by cell sorting of gradient-purified leukocytes according to the following surface markers. Plasmacytoid DCs: CD19-, CD3-, CD56-, HLA-DR+, CD11C-, CD14-, CD16-, CD304+. Myeloid CDs: CD19-, CD3-, CD56-, HLA-DR+, CD11C+, CD14-, CD16-, CD1c/CD141+/-. Classical monocytes: CD19-, CD3-, CD56-, HLA-DR+, CD11C+, CD14+, CD16- . Non-classical monocytes: CD19-, CD3-, CD56-, HLA-DR+, CD11C+, CD14lo, CD16+. THP1 and Hek293T cells were purchased from ATCC and cultured in RPMI 1640 (Thermo Fisher), 10 % FCS, 1% penicillin/streptomycin solution (Thermo Fisher). For BAY-11-7082 (NFκB inhibitor) and Ruxolitinib (JAK-STAT inhibitor) treatments, cells were pre-stimulated with the respective inhibitor or DMSO for 2 h prior to further stimulations. For PU.1 inhibition, cells were incubated with the inhibitor DB2313 (MedChemExpress) or DMSO for 4 h, followed by further sample processing. Cells were cultured at a density of 1 million cells per 2 ml culture medium in 6-well dishes or with evenly adjusted cell number and medium volume for smaller dishes. In all experiments, LPS was used at a concentration of 100 ng / ml, polyI:C at 10 µg / ml and Pam3CSK4 at 200 ng / ml. All cells were cultured at 37 °C in a humidified atmosphere with 5 % CO_2.

Patients suffering from SARS-CoV-2-infection were recruited after hospitalization. In addition, healthy subjects were recruited (Table S2 and 3). All COVID-19 patients were tested positive for SARS-CoV-2 RNA in nasopharyngeal swabs and graded to have mild (WHO 2-4) or severe (5-7) disease according to the WHO clinical ordinal scale. Immunosuppressed, pregnant and HIV-positive patients were excluded from the study. The BioInflame study was approved by the ethics committee of the Charité - Universitätsmedizin Berlin (EA2/030/09) and the University Medical Center Marburg (55/17). All blood donors were at least 18 years of age and provided written informed consent for use of their blood samples for scientific purposes. PBMCs were isolated by Pancoll gradient centrifugation of one collected Vacutainer EDTA-tube (6 ml whole blood). All methods were performed in accordance with the relevant guidelines and regulations.
Bronchoalveolar lavage (BAL) fluid (BALF) (Fig. 7F) was obtained at the University Clinics Giessen and Marburg (UKGM) (American Thoracic Society consensus procedure), on approval by the ethics committee (Marburg: 87/12). Late stage IPF tissue was obtained from the UGMLC Giessen Biobank/eurIPF registry biobank, member of the DZL Platform Biobanking, on approval by ethics committee (Az 58/15 and 111/08). The patients have been informed and given their written consent for the use of biospecimen for research purposes. Tissue was flushed with pre-warmed PBS. Obtained cells were analysed immediately. Further BALF (Fig. 7F) was obtained from patients at the Department of Infectious Diseases and Respiratory Medicine, Charité, Berlin. All patients underwent bronchoscopy including BAL on clinical indication and had provided oral and written informed consent. The study was approved by the local ethics committee (EA2/086/16). BAL was performed by instillation of 150 ml pre-warmed sterile 0.9% NaCl solution. In patients with focal abnormalities in chest imaging, BAL was performed in the corresponding pulmonary segment; in patients without radiological abnormalities or diffuse infiltrates, BAL was performed in the right middle lobe or lingula. Diagnosis of infection was made by a board-certified pulmonologist based on chest imaging, clinical signs of infection, culture and laboratory results, BALF cellular analysis and response to therapy. For the infection group, patients with non-mycobacterial infection were selected. Control patients showed no apparent lung disease and underwent bronchoscopy and BAL as part of rule-out diagnostics due to idiopathic coughing, for exclusion of pulmonary

involvement of systemic disease (e.g. rheumatoid arthritis) or for exclusion of pulmonary tuberculosis. No obvious abnormalities in chest imaging and BALF composition were detected in these patients. Patient characteristics are listed in Table S6. All studies and procedures to obtain human specimen were conducted according to the Declaration of Helsinki.

CRISPR/Cas9

PIRAT-deficient cells were generated by CRISPR/Cas9, as recently described (1), using independent gRNAs and the pX458 vector system (Fig. S9H). Control cells were generated using a pX458 vector with scrambled gRNA. For PU.1 and LUCAT1 silencing, a lentiviral CRISPR interference vector (2) was used (Addgene #71237). gRNAs targeting the PU.1 or LUCAT1 TSS were cloned into the vector followed by lentiviral particle production (see below) and transduction; transduced cells (GFP+) were purified by cell sorting (Aria III, BD) and lysed immediately (PU.1) or cultured (LUCAT1). GuideRNA sequences are provided in Table S7.

Lentiviral transduction

HEK293T cells were co-transfected with lentiviral vector, pseudotyping- and helper-plasmid (pVSVG and psPAX2) using lipofectamine 2000 (Thermo Fisher). For over-expression, the SparQ lentivector (Systembio, # QM511B-1) containing the RACE-refined PIRAT cDNA was used. Virus-containing supernatants were passed through a 0.45 µm filter. Cells were transduced by resuspension in virus containing supernatants and centrifugation at 37 °C and 800 g for 2 h. 48 h later, transduced cells were purified by cell sorting (Aria III, BD) based on GFP-expression.

qRT-PCR

RNA was isolated using TRIzol (Ambion), treated with DNaseI (Thermo Fisher) in the presence of recombinant RNase inhibitor (Promega) and concentration was determined (Nanodrop 2000 spectrometer, Thermo Scientific). cDNA was generated (High-Capacity cDNA Reverse Transcription Kit, Thermo Fisher) and quantitative PCR was performed (PowerUP SYBR Green Master Mix, Thermo Fisher) using a QuantStudio 3 instrument. For subcellular fractionation and CoIP analysis the Power SYBR RNA-to-Ct 1-Step Kit (Thermo Fisher) was used. Relative expression was calculated based on CT values, using the $2^{-\Delta\Delta CT}$ method (3), where applicable relative to U6 snRNA. Primers are listed in Table S7.

Subcloning and sequencing of REXO1LP amplicons

DNA from the elution fractions of PIRAT ChIRP experiments was used as a template for PCR reactions with the same primers as in Fig. S15A and using Advantage 2 proof-reading PCR polymerase (Takara). Experiments were conducted with ChIRP DNA from monocytes from three different blood donors. PCR products were subjected to gel-purification and sub-cloned using the Strataclone TA PCR cloning kit (Agilent). Insert sequences were determined by Sanger sequencing (Seqlab GmbH) and aligned using Multalin (http://multalin.toulouse.inra.fr/multalin/). Nucleotide variants in the sequenced inserts were counted and assigned to the three different donors.

RACE-PCR

RACE-PCR was performed using the SMARTer 5'/3' RACE kit (Clontech) according to the manufacturer's instructions. Template poly(A) RNA was purified using oligo-d(T) coupled dynabeads (Thermo Fisher). RACE-PCR primers are listed in Table S7. RACE products were subjected to gel-purification and sub-cloned using the Strataclone UA PCR cloning kit (Agilent). Insert sequences were determined by Sanger sequencing (Seqlab GmbH).

Copy number enumeration

PIRAT cDNA (Fig. S8) was amplified using Phusion PCR polymerase (Thermo Fisher), according to the manufacturer's instructions, and primers listed in Table S7. The forward primer (OBS-1898) contained a T7 RNA polymerase consensus binding site. The PCR amplicon was extracted from an agarose gel (Macherey-Nagel™ NucleoSpin™ Gel and PCR Clean-up Kit) and 150 ng were used as a template for RNA in vitro transcription (for 4 h), using the MEGAscript T7 transcription kit (Thermo Fisher), according to the manufacturer's instructions. Synthesized RNA was cleaned up using the Monarch RNA Cleanup column kit (NEB). RNA concentration was determined using the Agilent Bioanalyzer system, with an RNA Nano chip. RNA integrity was additionally controlled by running a sample on a MOPS / 1.2 % agarose / 1 % formaldehyde gel (10x MOPS buffer: 50 mM MOPS, 50 mM Na-acetate, 10 mM EDTA, pH 7.0), with Millennium RNA size marker (Thermo Fisher). PIRAT copy number was determined by qRT-PCR, as described in the main manuscript text.

Subcellular fractionation

Cells were lysed (10 mM Tris, pH 8, 140 mM NaCl, 1.5 mM $MgCl_2$, 0.5 % Igepal, 2 mM vanadyl ribonucleoside complex), incubated on ice for 5 min and centrifuged (1000 x g, 4 °C, 3 min). The supernatant (cytosolic fraction) was transferred to a new tube, centrifuged (3 min, maximum speed) and transferred to a new tube for RNA-extraction. The pellet (nuclear fraction) was washed two times with lysis buffer and once with lysis buffer containing 0.5 % deoxycholic acid (centrifugations at 4 °C and 1000 x g), followed by RNA-extraction.

RNA-FISH

Tissues were derived as described (4) with Charité University Medicine Berlin Ethics Committee approval no. EA2/079/13, formalin fixed, paraffin embedded and sectioned at 4 µm on glass slides. Probe sequences were designed by Affymetrix (Homo sapiens PIRAT (RUO) Catalog no. VA1-3025697; Homo sapiens EEF1A1 Catalog no. VA1-10418). RNA-FISH was performed using the ViewRNATM ISH Tissue 1-Plex Assay (Affymetrix) with heat pretreatment for 10 min and protease digestion for 20 min. A probe homologous to EF1α served as positive control for the hybridization conditions on consecutive tissue sections. Diluent without probe served as control for background staining. Roti®-Mount FluorCare DAPI (Carl Roth®) was used for counterstaining of nuclei and as mounting medium. Photographs were taken using an Olympus DP 80 microscope at 600x magnification (DAPI signal: 345 nm; red probe signals: 550 nm).

Western blot

Protein concentrations were determined using BCA (Pierce™ BCA Protein Assay Kit, ThermoFisher) and an Infinite PRO (Tecan) plate reader. Proteins were separated by SDS PAGE, using 10% polyacrylamide gels. Proteins were transferred onto a nitrocellulose membrane (Amersham™ Protran®, Sigma-Aldrich). For blot development and detection, the ECL Prime Western Blot Detection kit (Amersham) and a Chemostar Imager (INTAS Science Imaging) were used. Antibodies are listed in Table S8. Western blot full-scan is shown in Fig. S16A.

Flow cytometry

Cells were identified by plotting the respective fluorescence channel against background-fluorescence or the side-scatter. The gating strategy is illustrated in Fig. S11F. For surface marker staining, 2 µl of fluorophore-coupled primary antibody were added to cells in 100 µl PBS containing 1 % FCS, followed by incubation on ice for 30 min. Cells were washed and resuspended in PBS containing 0.5 % FCS and subjected to FACS analysis (Guava EasyCyte, Millipore).

ChIP

40 million cells per capture were crosslinked with PBS, 1 % formaldehyde for 10 min, quenched with 1/10th volume 1.25 M glycine for 5 min and resuspended in 800 µl lysis buffer (50 mM Tris-Cl, 10 mM EDTA, 1 % SDS, 1 mM PMSF). Lysate was sonicated (Diagenode Biorupter) until DNA appeared with a fragment size between 100 and 500 bp on agarose gels. Sample was adjusted with 3.6 ml ChIP Dilution Buffer (50 mM Tris-HCl, 0.167 M NaCl, 1.1% Triton X-100, 0.11% sodium deoxycholate), 2 ml RIPA-150 (50 mM Tris-HCl, 0.15 M NaCl, 1 mM EDTA pH8, 0.1% SDS, 1% Triton X-100, 0.1% sodium deoxycholate) and PMSF (1 mM). 60 µl of magnetic beads were coupled with PU.1 C1 + A7 antibody or FLAG antibody (Table S8), as described by Tawk et al. (5) and added to the diluted lysate, followed by rotation at 4 °C over-night. Upon one wash with RIPA-150, two washes with RIPA-500 (same as RIPA-150 but with 0.5 M NaCl), 2 washes with RIPA-LiCl (50 mM Tris-HCl, 1 mM EDTA pH 8, 1% Nonidet P-40, 0.7% sodium deoxycholate, 0.5 M LiCl2) and 2 washes with TE buffer (10 mM Tris-HCl, 1 mM EDTA), DNA input and bead samples were resuspended in 200 µl elution buffer (10 mM Tris-HCl, 0.3 M NaCl, 5 mM EDTA pH8, 0.5 % SDS). Until this step, all buffers were supplemented with cOmplete protease inhibitor (Roche). Following addition of 1 µl RNase A and incubation for 4 h at 65 °C beads were separated and supernatant was incubated with 10 µl of Proteinase K for 45 min at 50 °C. DNA was purified by PCI extraction and ethanol / sodium-acetate precipitation.

ChIRP

Antisense DNA probes (Table S9) were synthesized at Metabion AG and 3' mono-biotinylated using terminal transferse (New England Biolabs) and Biotin-11-ddUTP (Jena Bioscience) according to the manufacturer's instructions. ChIRP (20 million CD14+ monocytes per capture) was performed as described previously (6).

UV crosslinking & Co-immunoprecipitation

For co-immunoprecipitation (CoIP), 10^7 cells were UV-crosslinked (300 mJ / cm^2) in petri dishes, on an ice bath. The CoIP procedure published by Tawk et al. (5) was used with minor modifications. For protein purification protein G dynabeads (Thermo Fisher), coupled with 2.5 µg of antibody (Table S8) were used. In PU.1 CLIP experiments, eluate fractions were split up for protein analysis by Western blot and RNA extraction as described above.

Single Cell RNA-sequencing analysis

Single cell multiomics was performed using the BD Rhapsody system according to manufacturer's protocols. 250.000 PBMCs per sample (two patients and two healthy controls) were incubated with an individual oligo-labelled antibody (Multiplex Tag, BD Human Single-Cell Multiplexing Kit, Cat. No. 633781), for 20 minutes at room temperature. Cells were washed twice with BD Pharmingen Stain Buffer (Cat. No. 554656) and labelled cell suspensions were pooled and incubated with oligo-labelled AbSeq antibodies directed against CD206 (Cat. No. 940068), CD163 (Cat. No. 940058) and HLA-DR (Cat. No. 940010) for 30 minutes on ice. Upon two washes with Stain Buffer, cells were resuspended in Sample Buffer (Cat. No. 650000062) and viability-stained with 2 mM Calcein AM (Cat. No. C1430; Thermo Fisher Scientific, Dreieich, Germany) and 0.3 mM Draq7 (Cat. No. 564904) for 5 minutes at 37°C. The suspension was counted using a disposable hemocytometer (Cat. No. DHCN01-5; INCYTO, Cheonan, South Korea) and cell viability was determined.

The BD Rhapsody Cartridge (Cat. No. 400000847) was primed with 100% ethanol followed by 2 washes with Cartridge Wash Buffer 1 (Cat. No. 650000060) and one wash with Cartridge Wash Buffer 2 (Cat. No. 650000061). About 30.000 labelled cells were loaded and incubated for 15 minutes at room temperature. Excess fluid was removed and the cartridge was loaded with Cell Capture Beads (Cat. No. 650000089) and incubated for 3 minutes at room temperature. Excess

beads were washed off using Sample Buffer. Lysis Buffer was applied and beads were extracted from the cartridge using the BD Rhapsody Express instrument and washed twice with cold Bead Wash Buffer (Cat. No. 650000065).

The cDNA reaction mix was prepared as indicated in the manufacturer's protocol and mixed with the beads. The mixture was incubated in a thermomixer (37 °C, 1200 rpm, 20 minutes). The supernatant was removed and replaced by the Exonuclease I mix prepared according to the manufacturer's protocol. The bead suspension was placed on the thermomixer (37°C, 1200 rpm, 30 minutes, followed by 80 °C without shaking for 20 minutes). The suspension was then briefly placed on ice and the supernatant was removed. Finally, beads were resuspended in Bead Resuspension Buffer (Cat. No. 650000066).

Single cell mRNA, multiplex sample Tag, and AbSeq libraries were prepared using the BD Rhapsody™ Single-Cell Analysis system (Cat. No. 633774) according to manufacturer's recommendation (Doc ID: 214508). Briefly, the Bead Resuspension Buffer was removed from the beads and replaced by the PCR1 reaction mix containing the primers specific for the AbSeq and mutiplex sample tags, and genes of the Human Immune Response Panel (Cat. No. 633750) supplemented with custom-made primers for additional genes (see NCBI GEO GSE142503). Beads were placed in the thermal cycler for 11 cycles of the PCR program indicated in the protocol. The supernatant was retained and the PCR products for Abseq and multiplex sample tags, as well as the mRNA PCR product were separated and purified by double-sided size selection using AMPure XP magnetic beads (Cat. No. A63880; Beckman Coulter, Krefeld, Germany). A fraction of the Abseq/multiplex sample tag PCR 1 product, as well as the mRNA PCR 1 product were further amplified with a second PCR of 10 cycles and subsequent purification using AMPure XP beads, resulting in multiplex sample tag and mRNA PCR 2 product. Finally, the Abseq/multiplex sample tag PCR 1 product for the Abseq library, and both PCR 2 products for each the multiplex sample tag and mRNA libraries were amplified by the final index PCR for 7 cycles each with subsequent purification afterwards. Concentrations of the index PCR products were determined using the Qubit Fluorometer and the Qubit dsDNA HS Kit (Cat. No. Q32851; Thermo Fisher Scientific) and quality control was performed on the Agilent 2100 Bioanalyzer with the High Sensitivity DNA Kit (Cat. No. 5067-4626; Agilent, Waldbronn, Germany). Mixed libraries were sequenced on a NextSeq550 with 2 x 75 bp paired-end reads.

After pre-processing of BD Rhapsody scRNA-seq data, read counts were loaded into the R (v3.6.3) environment and further analyzed using the Seurat package (v3.1.4). The following quality criteria were used to include cells for the downstream analysis: at least 25 genes were expressed, and at least 1,000 but no more than 70,000 transcripts were detected per cell.

Following the Seurat workflow, the read counts were normalized and scaled by NormalizeData and ScaleData functions of the Seurat package, respectively. Principal component analysis (PCA) was performed by RunPCA using top 2,000 variable features that were selected using the default selection method ("vst") in Seurat. Next, based on the first 15 PCs, cell clusters were identified with the Louvain algorithm at resolution of 0.4. Finally, in a two-dimensional space, a UMAP was generated to visualize the identified cell clusters.

To identify marker genes of each cell cluster, differentially expressed (DE) genes were tested by FindAllMarkers functions in Seurat using the default test (Wilcoxon Rank Sum test). Significantly differentially expressed genes were determined by 1) log-fold changes > 0.3, 2) expressed in at least a fraction of 0.2 cells in each tested population, and 3) adjusted p value < 0.05 (Bonferroni correction). DE analyses were used to identify cluster marker genes by comparing the expression of upregulated genes in cells between one cluster and the rest of cells.

Cell clusters were firstly assigned using the SingleR (v1.0.6) package based on four reference dataset which are provided in the package, including BlueprintEncodeData, DatabaseImmuneCellExpressionData, HumanPrimaryCellAtlasData, and MonacoImmuneData. Then, the assigned cell cluster annotations were double-checked by comparing the cell type specifically expressed marker genes from public resources.
To dissect the different profiles between COVID-19 patients and controls, publicly reported COVID-19 related genes were selected and their expression profiles in patients and controls were

visualized for each identified cell cluster using a modified DotPlot function in Seurat. For PIRAT and S1000A8/A9 correlation analysis, the Spearman's correlation coefficient was calculated based on the gene expression across all cells and visualized using the R/ggplot2 package. P-value ≤ 0.05 was considered as a threshold for statistical significance.

Bulk sequencing and bioinformatics analysis

RNA was isolated (miRVana kit, Thermo Fisher) and DNaseI-digested as described above. RNA-quality was evaluated (Experion RNA analysis kit, BioRad) and Illumina TruSeq mRNA libraries were generated (Genomics Core Facility, Philipps-University Marburg), and analysed on a HiSeq 1500 machine. CLIP-seq and ChIRP-seq libraries were generated at Vertis Biotech AG (Germany) using in-house protocols and sequenced on a NexSeq500 device. Human Bodymap raw data (Fig. S1A) were obtained through European Nucleotide Archive (datasets ERR030888-ERR030903) (7). Peripheral blood leukocyte raw data (Fig. 1B) were downloaded from NCBI GEO (GSE62408 and GSE60424). ENCODE $CD14^{+}$-monocyte DNaseI-Seq, H3K4me3- and H3K27me3-ChIP-Seq data were downloaded from NCBI GEO (SRR608865, SRR608866, SRR568364, SRR568365, SRR568417, SRR568418) (8). NCBI data were extracted using the SRA toolkit. Haematopoietic lineage expression raw data were obtained through the Blueprint Consortium (EGAD00001000939, EGAD00001000919, EGAD00001000907, EGAD00001000922, EGAD00001001477, EGAD00001000675) (9).

Reads in fastq-format were quality-trimmed using the CLC genomics workbench, with standard settings. For genome-wide ChIRP-seq peak calling the CLC genomics workbench "Transcription Factor ChIP-Seq" module was used, with a P-value cut-off at 0.05 and the ChIRP-seq control datasets (control track in Fig. 6F) as background control. Resulting peaks were annotated using the "Annotate with Nearby Gene Information" module and GENCODE GRCh38 reference genome data. Results, including peak position and shape information are shown in Table S5. RNA-seq reads were mapped to the human GRCh38 reference (GENCODE), using the CLC genomics workbench. Gene expression changes were calculated using RPKMs (based on uniquely mapped reads). Genes with RPKMs < 0.5 under all experimental conditions in the respective RNA-seq dataset were excluded from further analysis. Hierarchical clustering was done using Cluster 3.0 (Eisen lab). Heatmaps were generated using JAVA TreeView (10). For pathway enrichment analysis and induced network analysis ConsensusPathDB (11) was used. For illustrating the overlap between PIRAT- and LUCAT1-controled genes (Fig. 5H), Cytoscape was used. For prediction of transcription factors (Fig. S12A), the ENCODE and ChEA Consensus TFs tab at Enrichr (https://maayanlab.cloud/Enrichr/) was used. For co-expression analysis (Fig. 1E) R^2 was calculated (Excel) based on RPKMs from RNA-seq dataset introduced in Fig. 1B, and ENSEMBL-IDs of genes with R^2 values ≥ 0.8 were analysed in ConsensusPathDB. PCA analysis was done based on row Z-scores, using the R-script prcomp (stats) with rgl package. Other plots were generated using GraphPad Prism, Excel or BoxPlotR (http://shiny.chemgrid.org/boxplotr/). Statistical analysis was performed using GraphPad Prism. Sequence conservation was determined using NCBI BLASTN and the major species reference genome, respectively. BLAST hits with ≥ 20 complementary nucleotides located within a genomic range of max. 100 kb were considered. ENSEMBL BLASTN was used in Fig. S15D. All obtained BLASTN-hits are shown in the ENSEMBL genome browser screenshot.

Supplementary References

1. Janga H*, et al.* (2018) Cas9-mediated excision of proximal DNaseI/H3K4me3 signatures confers robust silencing of microRNA and long non-coding RNA genes. *PloS one* 13(2):e0193066.
2. Thakore PI*, et al.* (2015) Highly specific epigenome editing by CRISPR-Cas9 repressors for silencing of distal regulatory elements. *Nature methods* 12(12):1143-1149.
3. Livak KJ & Schmittgen TD (2001) Analysis of relative gene expression data using real-time quantitative PCR and the 2(T)(-Delta Delta C) method. *Methods* 25(4):402-408.
4. Aznaourova M*, et al.* (2020) Noncoding RNA MaIL1 is an integral component of the TLR4-TRIF pathway. *Proceedings of the National Academy of Sciences of the United States of America* 117(16):9042-9053.
5. Tawk C, Sharan M, Eulalio A, & Vogel J (2017) A systematic analysis of the RNA-targeting potential of secreted bacterial effector proteins. *Sci Rep-Uk* 7.
6. Chu C, Quinn J, & Chang HY (2012) Chromatin isolation by RNA purification (ChIRP). *Journal of visualized experiments : JoVE* (61).
7. Illumina, Inc. 25861 Industrial Blvd Hayward, CA 94545, United States, Human Bodymap 2.0, *European Nucleotide Archive*, https://www.ebi.ac.uk/ena/browser/view/PRJEB2445?show=reads, data accessed on 2017-05-29.
8. The ENCODE Consortium, ENCODE epigenomic data, *NCBI Sequence Read Archive*, https://www.ncbi.nlm.nih.gov/bioproject/PRJNA63443, data accessed on 2017-08-16.
9. The Blueprint Consortium, Blueprint haematopoietic lineage data, *European Genome Phenome Archive*, https://ega-archive.org/studies/EGAS00001000284, https://ega-archive.org/datasets/EGAD00001000675, https://ega-archive.org/studies/EGAS00001000327, data accessed on 2019-06-19.
10. Saldanha AJ (2004) Java Treeview--extensible visualization of microarray data. *Bioinformatics* 20(17):3246-3248.
11. Kamburov A, Wierling C, Lehrach H, & Herwig R (2009) ConsensusPathDB--a database for integrating human functional interaction networks. *Nucleic acids research* 37(Database issue):D623-628.
